# Dynamic Hydroxyl–Yne Reaction with Phenols

**DOI:** 10.1021/acs.orglett.2c03518

**Published:** 2022-11-09

**Authors:** Tanausú Santos, Yaiza Pérez-Pérez, David S. Rivero, Raquel Diana-Rivero, Fernando García-Tellado, David Tejedor, Romen Carrillo

**Affiliations:** † Instituto Universitario de Bio-Orgánica Antonio González (IUBO), Universidad de La Laguna, P.O. Box 456, 38206 La Laguna, Tenerife, Spain; ‡ Instituto de Productos Naturales y Agrobiología (IPNA-CSIC), Avda. Astrofísico Fco. Sánchez 3, 38206 La Laguna, Spain

## Abstract

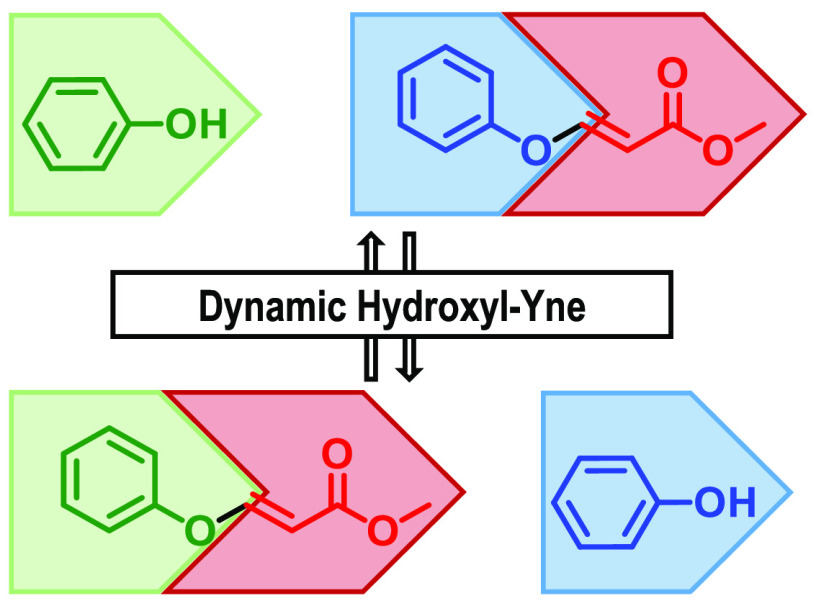

Dynamic Covalent Chemistry (DCvC) has gained increasing importance in supramolecular chemistry and materials science. Herein we prove the dynamic nature of the exchange between phenols and vinyl ethers. Exchange is fast at room temperature and under mild conditions. The equilibrium constants and the electronic effect of the phenol substituents were calculated. This novel incorporation to the DCvC toolbox could be quite useful, and as a proof it was used for the synthesis of a responsive molecular cage.

Reversible reactions confer to any chemical system the ability of adapting and responding to changes in the medium.^1^ In such a way, they mimic the adaptability of supramolecular systems,^2^ but with (usually) more robust covalent structures.^3^ During the last two decades Dynamic Covalent Chemistry (DCvC) has been developed, taking advantage of reversible reactions to generate controlled molecular systems and networks, designed self-processes, and complex architectures.^4−6^ In spite of a growing number of new reactions included in the DCvC toolbox,^7−11^ there is still a limited set of dynamic reactions, which impedes the flourishing of novel functions and applications. Additionally, useful reactions for DCvC should ideally not only be reversible but also efficient, employing common functional groups and displaying high atom economy.

In this regard, the 1,4-conjugate addition reaction between a suitable nucleophile and an activated alkyne is a highly versatile click-like process with a complete atom economy.^12^ The dynamic nature of these reactions has only been reported for the thiol–yne and amino–yne reactions (Scheme 1a,b). Indeed, several examples of dynamic thiol–yne reactions have been reported^13^ and applied to the synthesis of dynamic polymers.^14−17^ Although less explored than its thiol counterpart, the dynamic amino–yne reaction has recently grown considerable interest,^18,19^ particularly in the polymer community.^20,21^

**Scheme 1:**
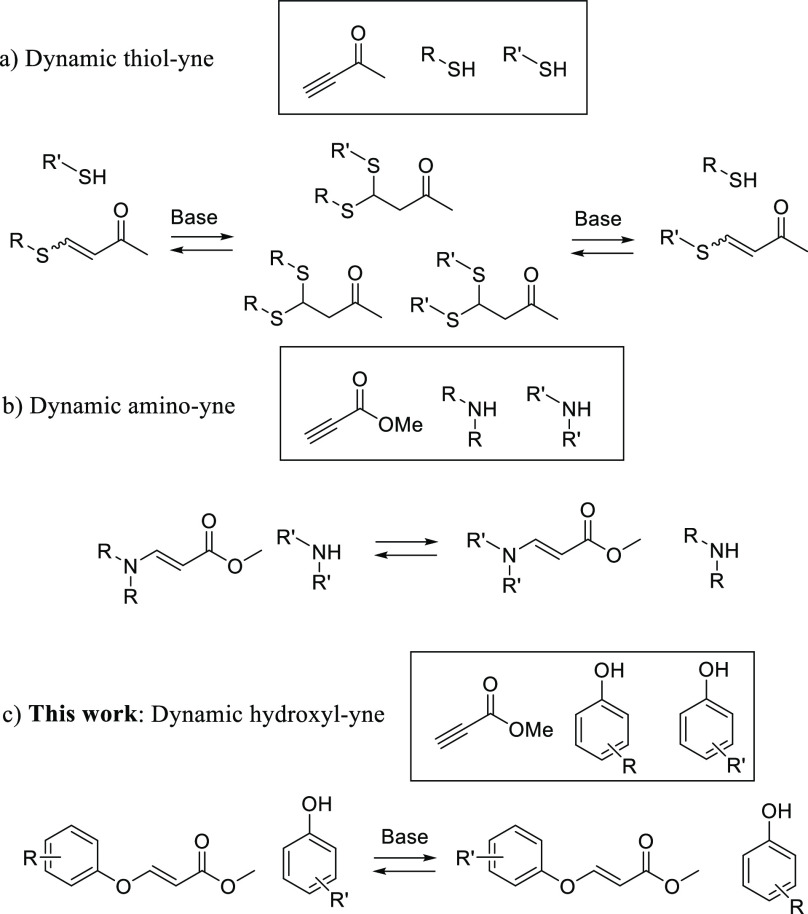
Dynamic 1,4-Conjugate Addition Reactions to Activated Alkynes

In spite of the proven potential of the thiol and amino–yne reactions, they display some drawbacks. Indeed, the thiol–yne reaction shows no stereoselectivity and the alkene is obtained almost invariably as a mixture of *E*/*Z* isomers; usually it is also difficult to stop in the monoaddition. On the other hand, the amino–yne reaction is, by definition, noncompatible with one of the most commonly used dynamic reactions such as imine exchange. The hydroxyl–yne reaction would have none of the above-mentioned problems, and yet, its reversibility has never been studied (Scheme 1c), even when interesting precedents suggest such reversibility.^23^ Indeed, Vilarrasa et al. reported a protecting group for catechols inspired in the double Michael addition to propiolate esters. They described translocations of the protecting groups between two different OH, which could only be explained by a dynamic behavior.

Herein, we prove the dynamic nature of the exchange between phenols and vinyl ethers by obtaining the same distribution of compounds in a series of reactions conducted in both forward and reverse directions (Figure 1a). The exchange could be optimized to be fast at room temperature. An appropriate scope of the reaction was examined, and equilibrium constants were calculated for all of the examples reported.

**Figure 1:**
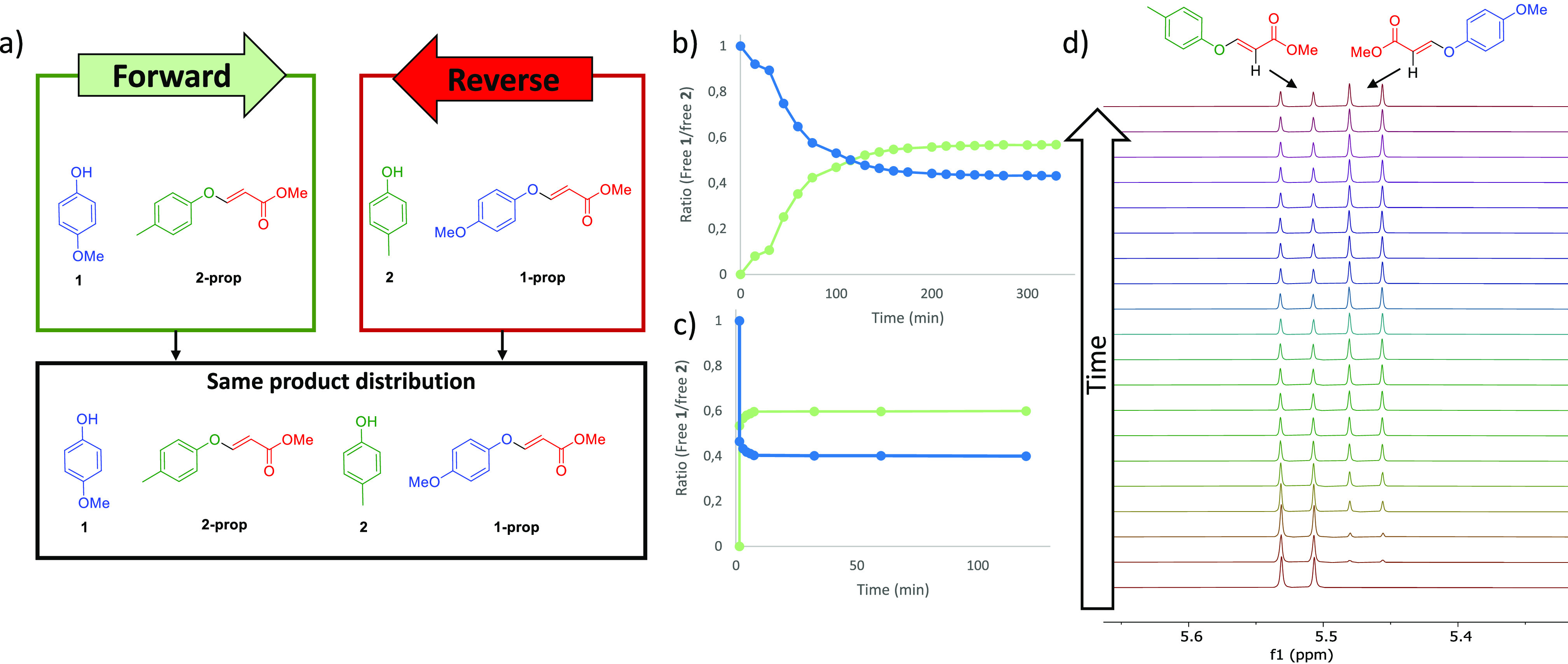
(a) Dynamic exchange of phenols starting from one end of the equilibrium (forward direction) or the other (reverse direction) yields the same distribution of compounds. An identical result is obtained even if reaction is carried out from the starting materials (neutral direction). (b) Kinetics of the forward reaction with DMAP in DMSO at 90 °C, following the ratio of free phenol **1** (blue) vs free phenol **2** (green).^22^ (c) Kinetics of the forward reaction with Cs_2_CO_3_ in DMSO at 25 °C, following the ratio of free phenol **1** (blue) vs free phenol **2** (green). (d) Stacked plot of ^1^H NMR of the forward reaction with DMAP at 90 °C recorded at different times.

The reversibility of the hydroxyl–yne reaction was initially tested with the system shown in Figure 1a: the forward direction was defined as the exchange between an equimolar mixture of *p*-methoxyphenol (**1**) and the vinyl ether **2-prop**, while the reverse reaction was defined as the exchange between *p*-cresol (**2**) and vinyl ether **1-prop**. A truly dynamic process would yield the same distribution of compounds regardless of whether the reaction was conducted in the so-called forward or reverse directions. Experimentally, it could be qualitatively confirmed by obtaining indistinguishable NMR spectra for reactions conducted in both directions. Obviously, the hydroxyl–yne exchange requires a base to improve the phenol nucleophilicity. The choice of the base proved to be a key factor in this dynamic reaction (Table 1).

**Table 1:** Influence of the Base and the Solvent in the Dynamic Hydroxyl–Yne Reaction

Entry	Base^a^	Solvent	Temp (°C)	Equilibration Time^b^
1	TEA^d^	DMSO-*d*_6_	90	n.r.^c^
2	DABCO^e^	DMSO-*d*_6_	90	>48 h
3	DBU^f^	DMSO-*d*_6_	90	decomposition
4	PBu_3_^g^	DMSO-*d*_6_	90	n.r.
5	DMAP^h^	DMSO-*d*_6_	90	200 min
6	DMAP	CD_3_CN	82	>48 h
7	DMAP	CDCl_3_	61	n.r.
8	K_2_CO_3_	DMSO-*d*_6_	25	12 h
9	Cs_2_CO_3_	DMSO-*d*_6_	25	10 min
10	Cs_2_CO_3_	CD_3_CN	25	4 h
11	Cs_2_CO_3_	CDCl_3_	25	>48 h
12	−i	DMSO-*d*_6_	90	n.r.

a 2 equiv of base was used.

b Equilibration time was calculated when no more changes in the NMR spectra were detected, and it was identical in both directions.

c n.r.: No reaction is observed after 1 week.

d Triethylamine.

e 1,4-Diazabicyclo[2.2.2]octane.

f 1,8-Diazabicyclo[5.4.0]undec-7-ene.

g Tributylphosphine.

h 4-Dimethylaminopyridine.

i No base added.

Many N-bases were not able to promote the reaction or they promoted it quite slowly, such as triethylamine or DABCO, while DBU led to decomposition (entries 1–3), even when all of them have been reported to efficiently catalyze the Michael addition to propiolates.^12,24^ Phosphines do not promote the hydroxyl–yne exchange (entry 4).^25^ Fortunately, the reaction reached equilibrium in roughly 3 h with DMAP in DMSO-*d*_6_ at 90 °C (entry 5). In a less polar solvent such as CD_3_CN, the reaction was very slow even at reflux for days (entry 6), and in CDCl_3_ it did not proceed (entry 7). The reaction temperature could be reduced by using an inorganic base such as potassium carbonate: the equilibrium is reached in DMSO-*d*_6_ at room temperature in a matter of hours (entry 8). The higher solubility of cesium carbonate in DMSO-*d*_6_ reduced the equilibration time to 10 min at 25 °C (entry 9). A comparison of the kinetics of the forward reaction with DMAP (90 °C) and Cs_2_CO_3_ (25 °C) can be seen in Figure 1c,d, respectively. In less polar solvents, such as deuterated acetonitrile (entry 10), exchange with Cs_2_CO_3_ is slightly slower, while in CDCl_3_ the exchange is dampened (entry 11). In all cases, <5% of the *Z* isomers is detected.

Remarkably, the dynamic behavior is observed even when reactions are submitted directly from the starting materials, i.e., an equimolar mixture of methyl propiolate and both phenols, which initially generates a kinetic mixture of vinyl ethers and phenols, and they rapidly equilibrate.^26^ On the other hand, alkyl alcohols are not able to undergo the dynamic exchange reaction, not even by heating or with the use of stronger bases.^27^

In order to explore how the outcome of the equilibria is affected by the electronic effect or the orientation of different substituent in the phenol, the optimized conditions (Cs_2_CO_3_ at 25 °C) were applied to several phenols in DMSO-*d*_6_ (Figure 2). In all cases, reversibility of the reactions was confirmed. Once in the equilibrium, all the compounds in the reaction mixture were quantified by ^1^H NMR to obtain the molar ratio of each of them, and straightforwardly the equilibrium constant could be calculated. In this regard, there are surprisingly very few papers in which equilibrium constants of the exchange of a dynamic covalent reaction are calculated.^28−30^ In order to ease the interpretation of the data, we compared all of the examples to *p*-methoxyphenol **1** (Figure 2a). It is evident from the results that electron-rich phenols tend to remain attached as ether, while electron-deficient phenols tend to be released (Figure 2b). Indeed, the *p*-OMe group favors the formation of the corresponding vinyl ether, while electron-withdrawing groups such as *p*-Cl and particularly *p*-COOMe induce the release of the phenol to remain free in solution. Actually, an excellent correlation was found between the equilibrium constant and the Hammett σ_p_ (Figure 2c).^31^ The slope of the regression line (ρ > 1) is probably related to an associative mechanism of exchange: addition of a phenol to the vinyl ether, generating a negatively charged enolate in the rate-determining step, followed by elimination of one of the phenols. Unfortunately, we were not able to detect such intermediate.

**Figure 2:**
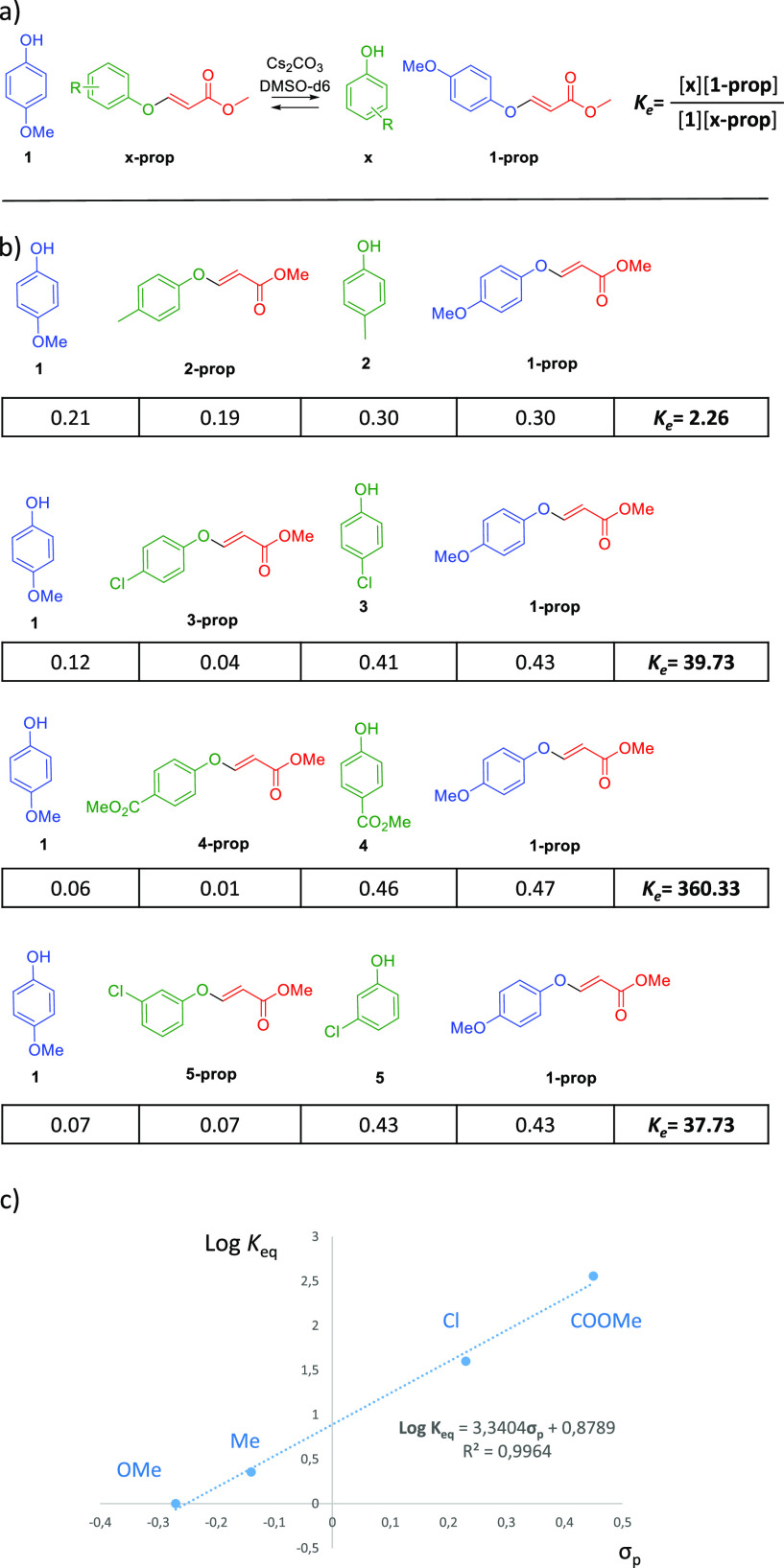
(a) Different phenols were tested against **1** to evaluate the effect of the substituent in the phenol. (b) Equilibria studied. For each of them, molar fraction of all compounds and the equilibrium constants were measured. (c) Correlation of the Hammett’s σ_p_ with the log *K*_eq_.

Other activated alkynes were tested to check the scope of the reaction (Figure 3a). An equimolar mixture of *p*-methoxyphenol (**1**), *p*-cresol (**2**), and the corresponding alkyne with 2 equiv of Cs_2_CO_3_ in DMSO-*d*_6_ was followed by ^1^H NMR (Figure 3b). Obviously, other propiolate esters, such as ethyl propiolate, yielded similar results. Internal alkynes also led to a successful exchange, as well as secondary amides, although in both cases the kinetics are much slower (2 and 5 days, respectively). It is worth mentioning that the amide yielded mostly *Z* isomer (∼70%). Phenyl ethynyl ketone (**keto**) and primary amides (**propNprim**) led to a successful exchange (Figure 3c), although progressive degradation to unknown products is observed in the conditions of the reaction, which precludes a reliable quantification.^27^

**Figure 3:**
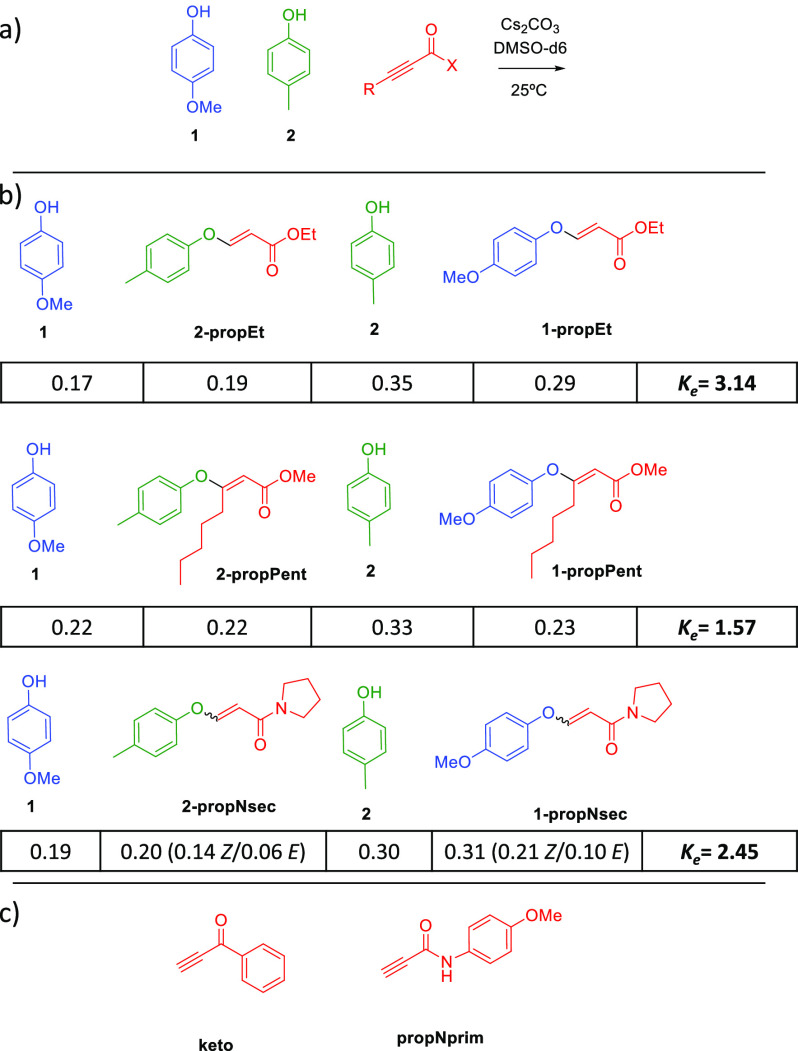
(a) Different alkynes were reacted with **1** and **2** under the optimized conditions. (b) Scope of activated alkynes. (c) Ketone and primary amide chosen for these experiments.

The compatibility of the dynamic hydroxyl–yne reaction with the commonly used imine exchange was tested. Under the optimized conditions, in a one-pot procedure (Scheme 2), 4-aminophenol **6** underwent O-addition to methyl propiolate, and subsequently, the remaining amino group could slowly react with an aldehyde to yield the corresponding imine **8**.^27^

**Scheme 2:**
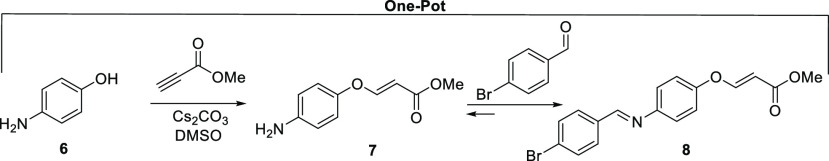
Compatibility of Hydroxyl–Yne Reaction with Imine Exchange

Finally, to prove the applicability of this exchange, we synthesized the responsive hemocryptophane **(±)11** in one step and moderate yield from cyclotriveratrylene derivative **(±)9**(32) and conformationally restricted tripropiolate **10** (Scheme 3). As expected, cage **11** is thermodynamically quite stable, but under an excess of phenol **1**, it is disassembled.^27^

**Scheme 3:**
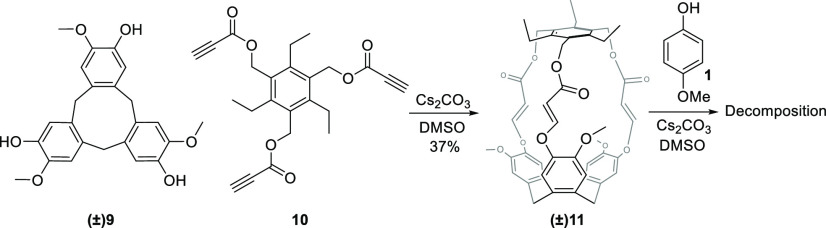
Synthesis and Disassembly of a Responsive Cage

In conclusion, the dynamic nature of the hydroxyl–yne reaction with phenols has been confirmed. The conditions for the exchange were optimized, and the reaction can be performed at room temperature with carbonate as base. The scope of the reaction with different phenols and activated alkynes was determined, and a quantification of all the species in equilibrium in every single example was carried out. The equilibrium constants could be calculated as well as the influence of the electronic nature of the substituent, which is correlated to the corresponding Hammett’s sigma. This reaction is compatible with other dynamic processes such as the imine exchange. Finally, the versatility of this dynamic click-like reaction was proven by the synthesis of a responsive molecular cage.

## Data Availability

The data underlying this study are available in the published article and its online Supporting Information.

## Supporting Information Available

Experimental procedures, quantification by NMR, kinetic studies (PDF)
