# Supplementary material for: Dynamic Hydroxyl–Yne Reaction with Phenols
Source: Org Lett. 2022 Nov 9;24(45):8401–5. doi: 10.1021/acs.orglett.2c03518 (PMC10443044; doi:10.1021/acs.orglett.2c03518)
Supplement: Supplementary file 1 — ol2c03518_si_001.pdf [file ol2c03518_si_001.pdf]

SUPPORTING INFORMATION

DYNAMIC HYDROXYL-YNE REACTION WITH PHENOLS

Tanausú Santos,[†]* Yaiza Pérez-Pérez,[‡] David S. Rivero,[‡] Raquel Diana-Rivero,[‡] Fernando García-Tellado,[‡] David Tejedor,[‡]* Romen Carrillo[‡]*

[†] *Instituto Universitario de Bio-Orgánica Antonio González (IUBO), Universidad de La Laguna, P.O. Box 456, 38206 La Laguna, Tenerife, Spain.*

[‡]*Instituto de Productos Naturales y Agrobiología (IPNA-CSIC), Avda. Astrofísico Fco. Sánchez 3, 38206 La Laguna, Spain.*

rcarrillo@ipna.csic.es

dtejedor@ipna.csic.es

tsantoss@ull.edu.es

Table of Contents

1. Materials and methods S2
2. General procedure for the synthesis of vinyl ether derivatives S2
3. Characterization and spectral data S2
4. Covalent Dynamic Studies: ^{1}H NMR experiments. S4
5. Quantification and Equilibrium Constants. S5
6. Equilibria in CD_3CN. S19
7. Kinetic Studies S20
8. Reaction with ketones and primary amides S27
9. Attempts of dynamic hydroxyl-yne with alkyl alcohols S31
10. Compatibility of the hydroxyl-yne reaction with the imine exchange S32
11. Synthesis of hemicryptophane (±)11 and disassembly studies S32
12. NMR spectra S35
13. References S41

Materials and methods.

All reagents from commercial suppliers were used without further purification. All solvents were freshly distilled before use from appropriate drying agents. All other reagents were recrystallized or distilled when necessary. Analytical TLCs were performed with silica gel 60 F_{254} plates. Visualization was accomplished by UV light or vanillin with acetic and sulfuric acid in ethanol with heating. Column chromatography was carried out using silica gel 60 (230-400 mesh ASTM). ^{1}H NMR spectra were recorded at 500 MHz and 400MHz, ^{13}C NMR spectra were recorded at 126 MHz and 100 MHz. Chemical shifts were reported in units (ppm) by assigning TMS resonance in the ^{1}H NMR spectrum as 0.00 ppm (deuterated chloroform, 7.26 ppm; acetonitrile-*d*$_3$ 1.94 ppm; DMSO-*d*$_6$ 2.50 ppm). Data were reported as follows: chemical shift, multiplicity (s = singlet, d = doublet, t = triplet, q=quartet, dd = double doublet, ddd = double double doublet, m =multiplet and br = broad), coupling constant (*J* values) in Hz and integration. Chemical shifts for ^{13}C NMR spectra were recorded in ppm from tetramethylsilane using the central peak of $CDCl_3$ (77.14 ppm) as the internal standard. High resolution mass spectra (HRMS) was measured by ESI method with an Agilent LC-Q-TOF-MS 6520 spectrometer.

General procedure for the synthesis of vinyl ether derivatives

To a solution of the appropriated phenol (1.0 mmol, 1 equiv.) in CH_2Cl_2 (10 mL) were added DABCO (1,4-Diazabicyclo[2.2.2]octane) (0.1 mmol, 0.1 equiv.) and methyl propiolate (1.1 mmol, 1.1 equiv.) dropwise. The reaction mixture was stirred at room temperature for 1 hour. Then, the solvent was removed under vacuum and the crude was purified by silica gel flash column chromatography using an elution of ethyl acetate/hexane to afford the desired product.

Characterization and spectral data.

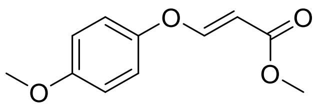

methyl (E)-3-(4-methoxyphenoxy)acrylate (1-prop). Purified by silica gel chromatography (EtOAc/Hexane: 10:90 v /v). clear oil (171 mg, 82%).**^{1}H NMR** (400 MHz, $CDCl_3$) δ 7.75 (d, *J* = 12.2 Hz, 1H), 7.03 – 6.95 (m, 2H), 6.92 – 6.84 (m, 2H), 5.46 (d, *J* = 12.2 Hz, 1H), 3.80 (s, 3H), 3.72 (s, 3H).**^{13}C NMR** (101 MHz, $CDCl_3$) δ 167.9, 160.6, 157.0, 149.7, 119.5, 115.0, 101.0, 55.8, 51.4. **HR-MS** (ESI$^+$, m/z): $[M+H]^+$= $C_{11}H_{13}O_4$, calcd.: 209.0814; found 209.0811.

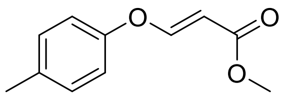

methyl (E)-3-(p-tolyloxy)acrylate (2-prop). Purified by silica gel chromatography (EtOAc/Hexane: 5:95 v /v). clear oil (152mg, 78%).**^{1}H NMR** (400 MHz, $CDCl_3$) δ 7.78 (d, *J* = 12.2 Hz, 1H), 7.19 – 7.13 (m, 2H), 6.98 – 6.92 (m, 2H), 5.52 (d, *J* = 12.2 Hz, 1H), 3.72 (s, 3H), 2.34 (s, 3H).**^{13}C NMR** (101 MHz, $CDCl_3$) δ 167.9, 159.9, 153.9, 134.8, 130.5, 118.0, 101.4, 51.4, 20.9. **HR-MS** (ESI$^+$, m/z): $[M-OCH_3]^+$= $C_{10}H_9O_2$, calcd.: 161.0603; found 161.0598.

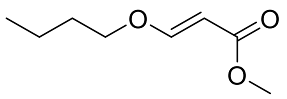

methyl (E)-3-butoxyacrylate. Purified by silica gel chromatography (EtOAc/Hexane: 5:95 v /v). clear oil (105 mg, 66%).**^{1}H NMR** (400 MHz, $CDCl_3$) δ 7.60 (d, *J* = 12.6 Hz, 1H), 5.19 (d, *J* = 12.7 Hz, 1H), 3.84 (t, *J* = 6.5 Hz, 2H), 3.70 (s, 3H), 1.74 – 1.62 (m, 2H), 1.48 – 1.34 (m, 2H), 0.94 (t, *J* = 7.4 Hz, 3H).**^{13}C NMR** (101 MHz, $CDCl_3$) δ 168.5, 162.9, 96.1, 71.1, 51.21, 31.0, 19.1, 13.8. **HR-MS** (ESI^{+}, m/z): $[M+H]^{+}$= $C_8H_{15}O_3$, calcd.: 159.1021; found 159.1018.

Covalent Dynamic Studies: ^{1}H NMR experiments.

General procedure

To a solution of the corresponding phenols (0.081 mmol of each one, 1 equiv.), and the corresponding activated alkyne (0.081 mmol, 1 equiv.) in DMSO-*d*$_6$ as a deuterated solvent (0.5 mL) was added Cs_2CO_3 (0.163 mmol, 2 equiv.). The reaction mixture was monitored by ^{1}H NMR at 25°C until thermodynamic equilibrium was reached. The last spectrum was selected for the calculation of the equilibrium constants. NMR spectra was processed and analyzed using Bruker Topspin 4.1 and MNova software. Peak areas were calculated by integration and/or using line-fitting to a Lorentz-Gauss functions using routines incorporated in the previously mentioned software.

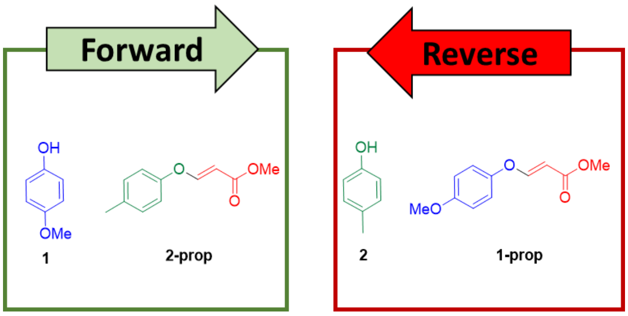

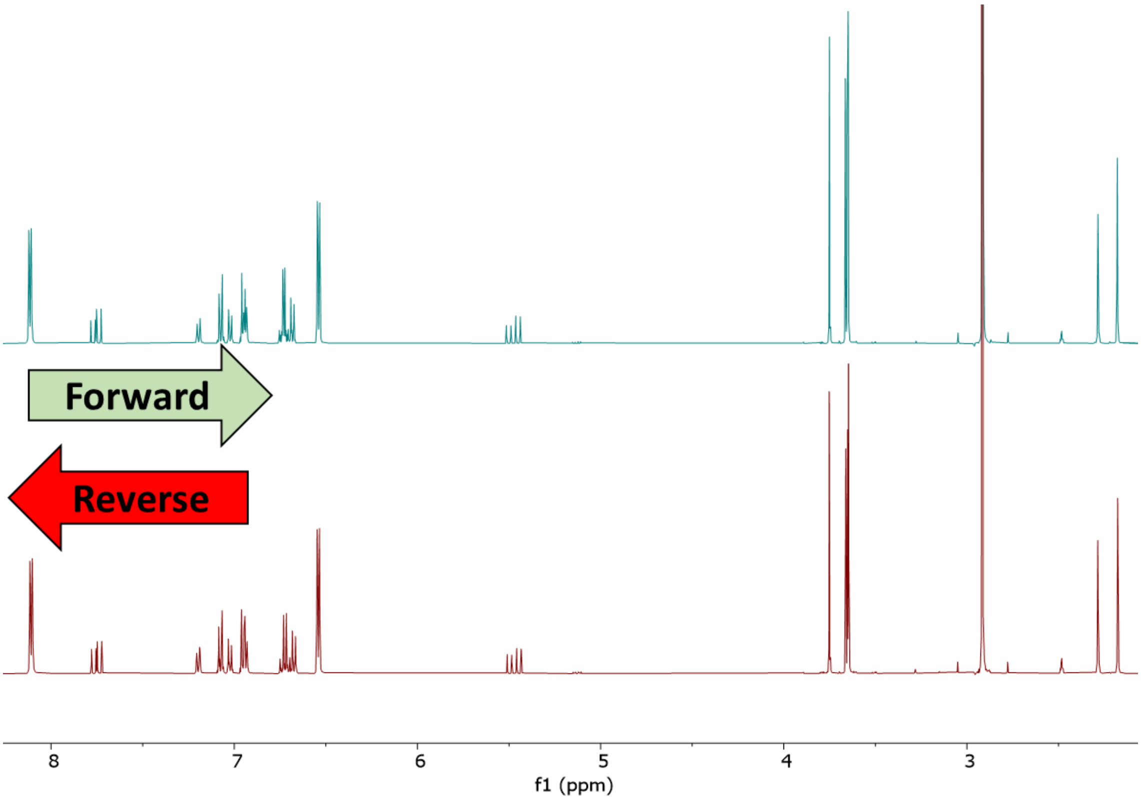

DMSO-*d*$_6$ (400 MHz)

Quantification and Equilibrium Constants.

System 1

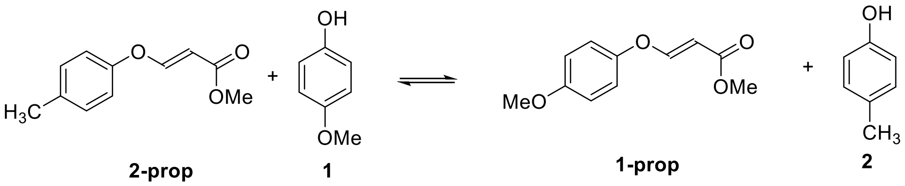

$$K = \frac{[2][1-\text{prop}]}{[1][2-\text{prop}]}$$

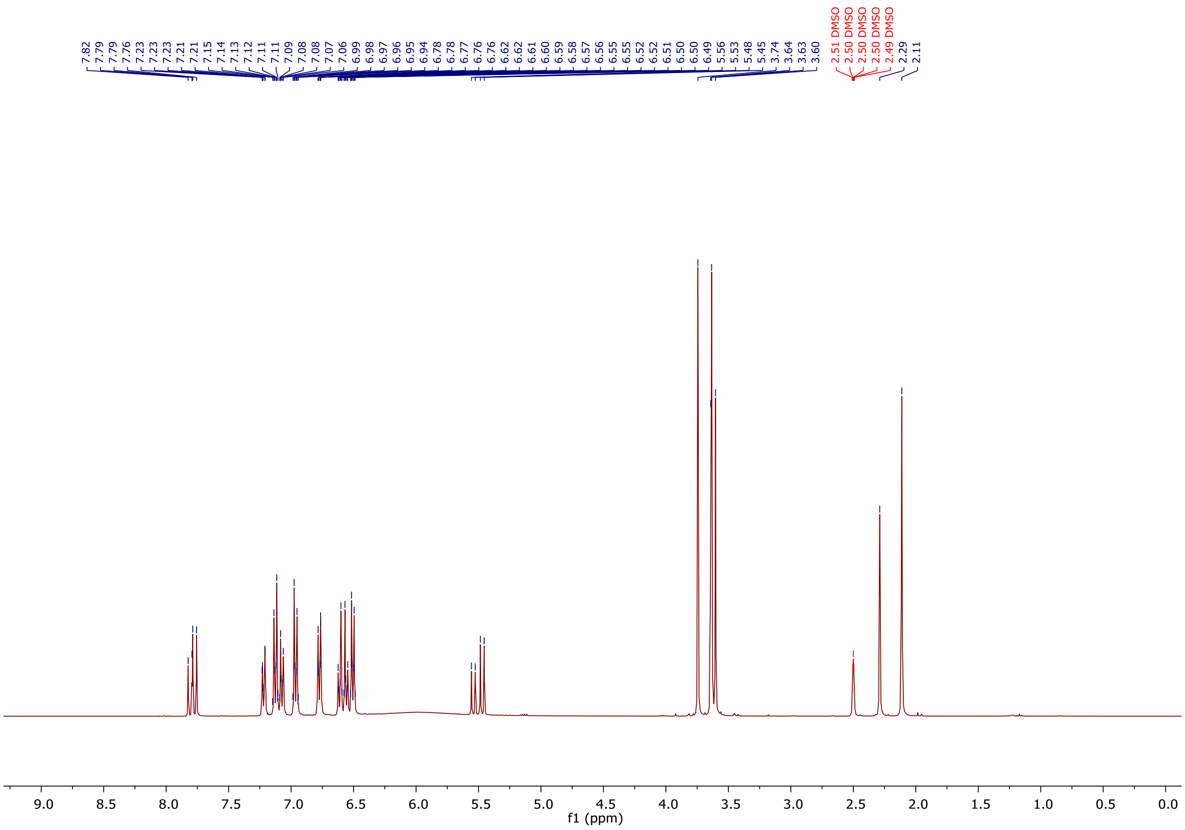

DMSO-d_6 (400 MHz)

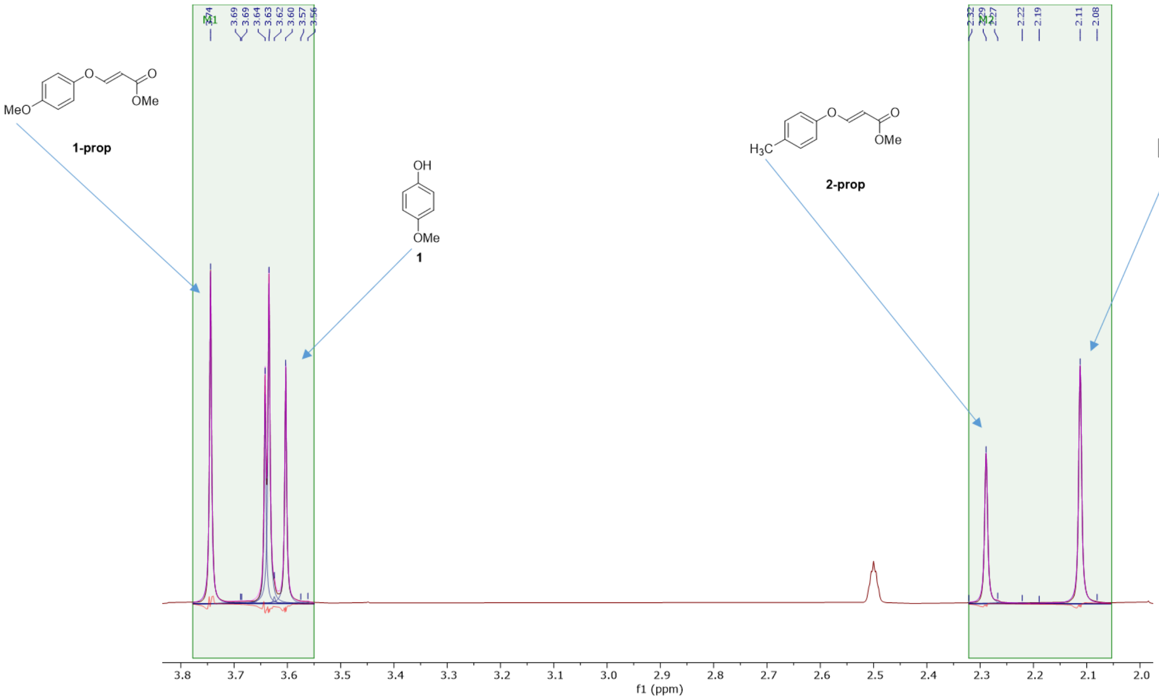

Compound	ppm	Area	Molar Fraction
1	3.60	303429076.32	0.21
2-prop	2.29	277747574.66	0.19
2	2.11	428302623.42	0.30
1-prop	3.74	420061611.26	0.30

$$K = \frac{[0.30][0.30]}{[0.21][0.19]} = 2.26$$

System 2

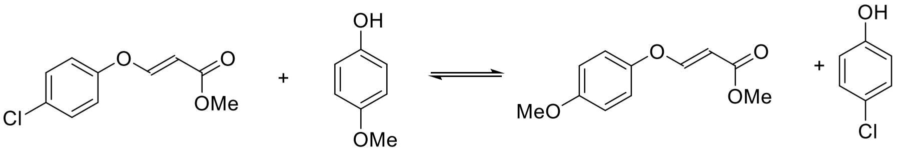

3-prop **1** **1-prop** **3**

$$K = \frac{[3][1-\text{prop}]}{[1][3-\text{prop}]}$$

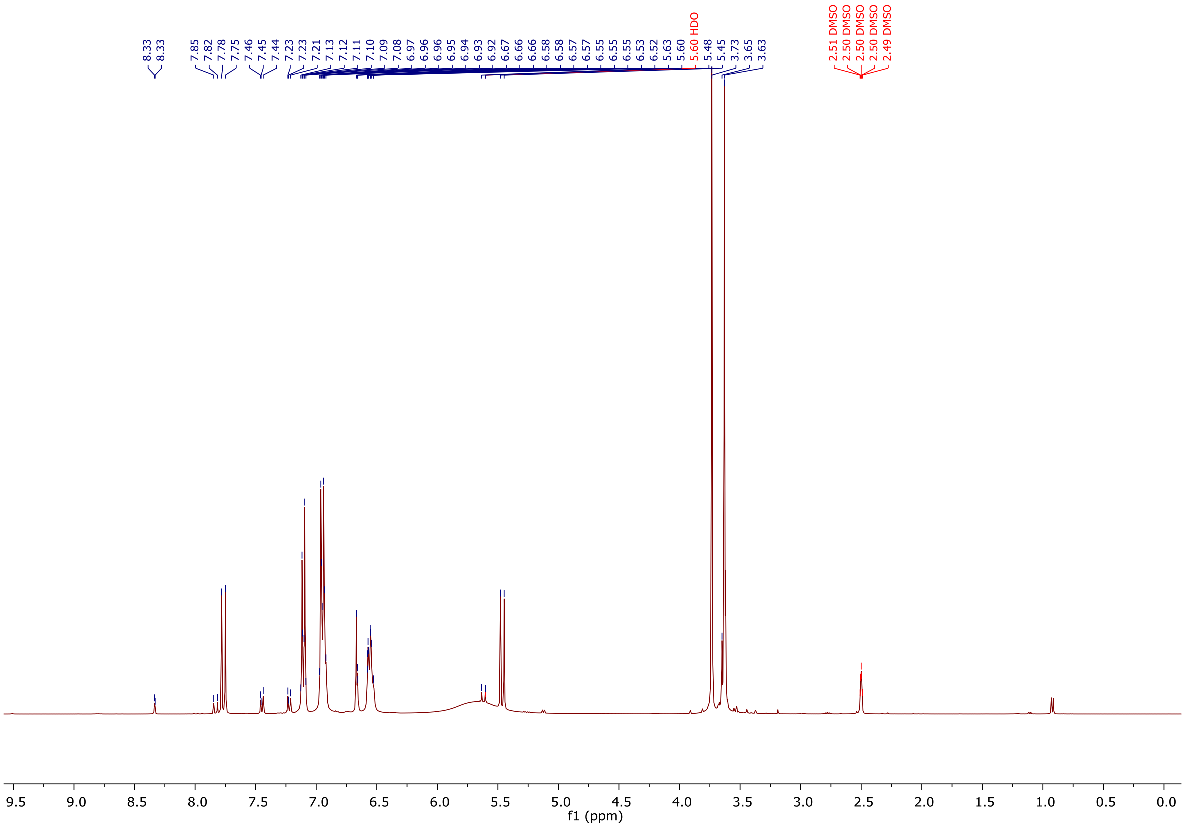

DMSO-d_6 (400 MHz)

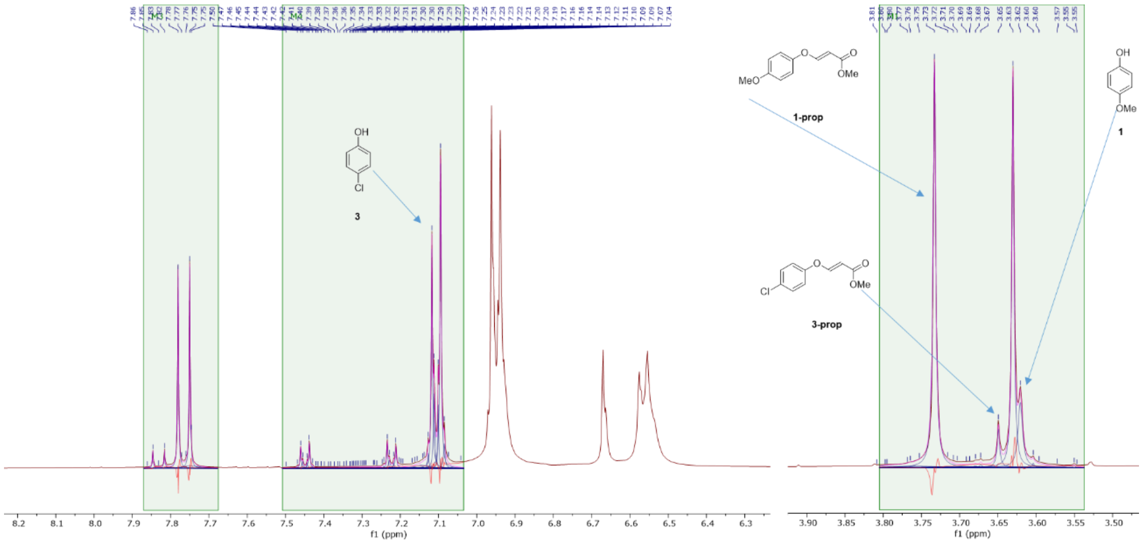

Compound	ppm	Area	Relative area[a]	Molar Fraction
1	3.62	205735373.97	68578457.99	0.12
3-prop	3.65	70692275.56	23564091.85	0.04
3	7.10	472452228.40	236226114.20	0.41
1-prop	3.73	752897583.32	250965861.1	0.43

[a] Relative area is calculated by dividing the total area by the number of equivalent hydrogens.

$$K = \frac{[0.41][0.43]}{[0.12][0.04]} = 39.73$$

System 3

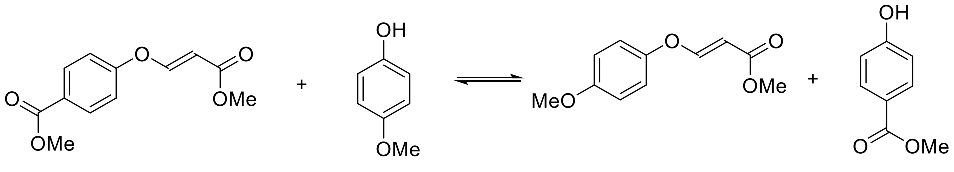

$$K = \frac{[4][1-\mathrm{prop}]}{[1][4-\mathrm{prop}]}$$

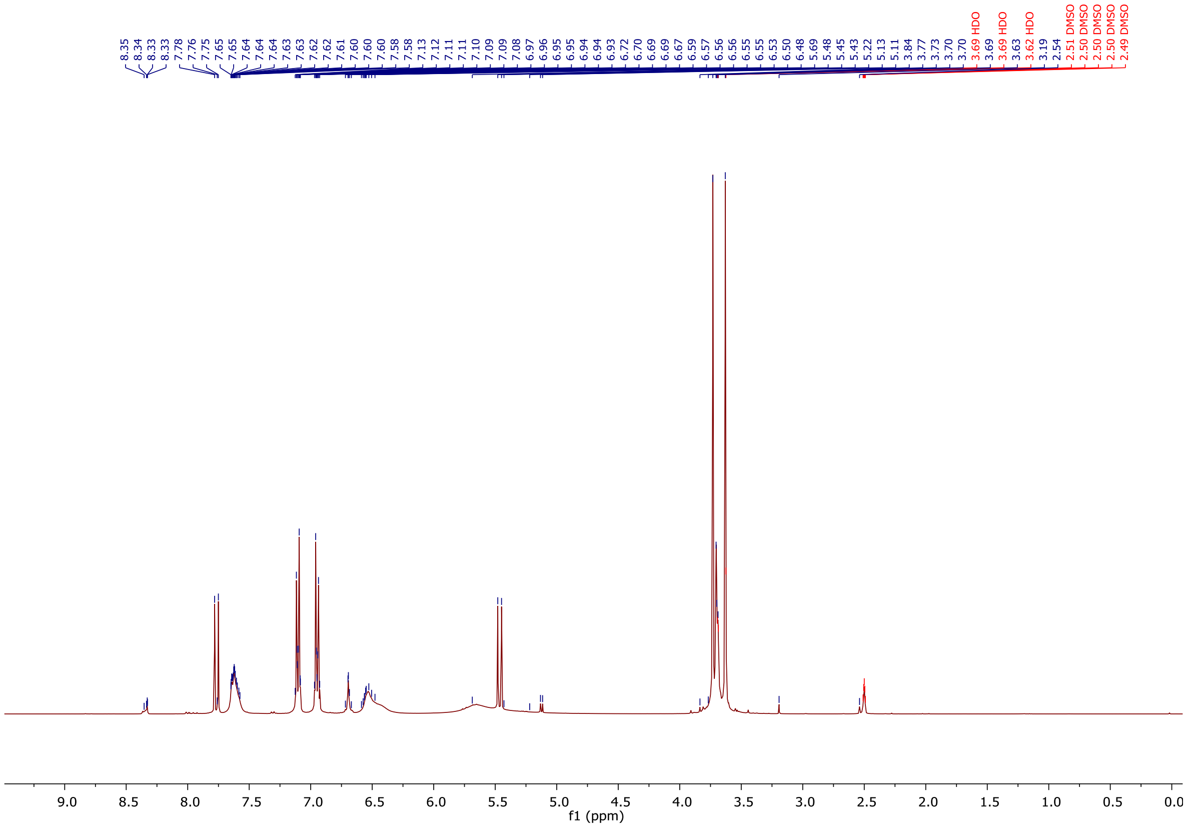

DMSO-d_6 (400 MHz)

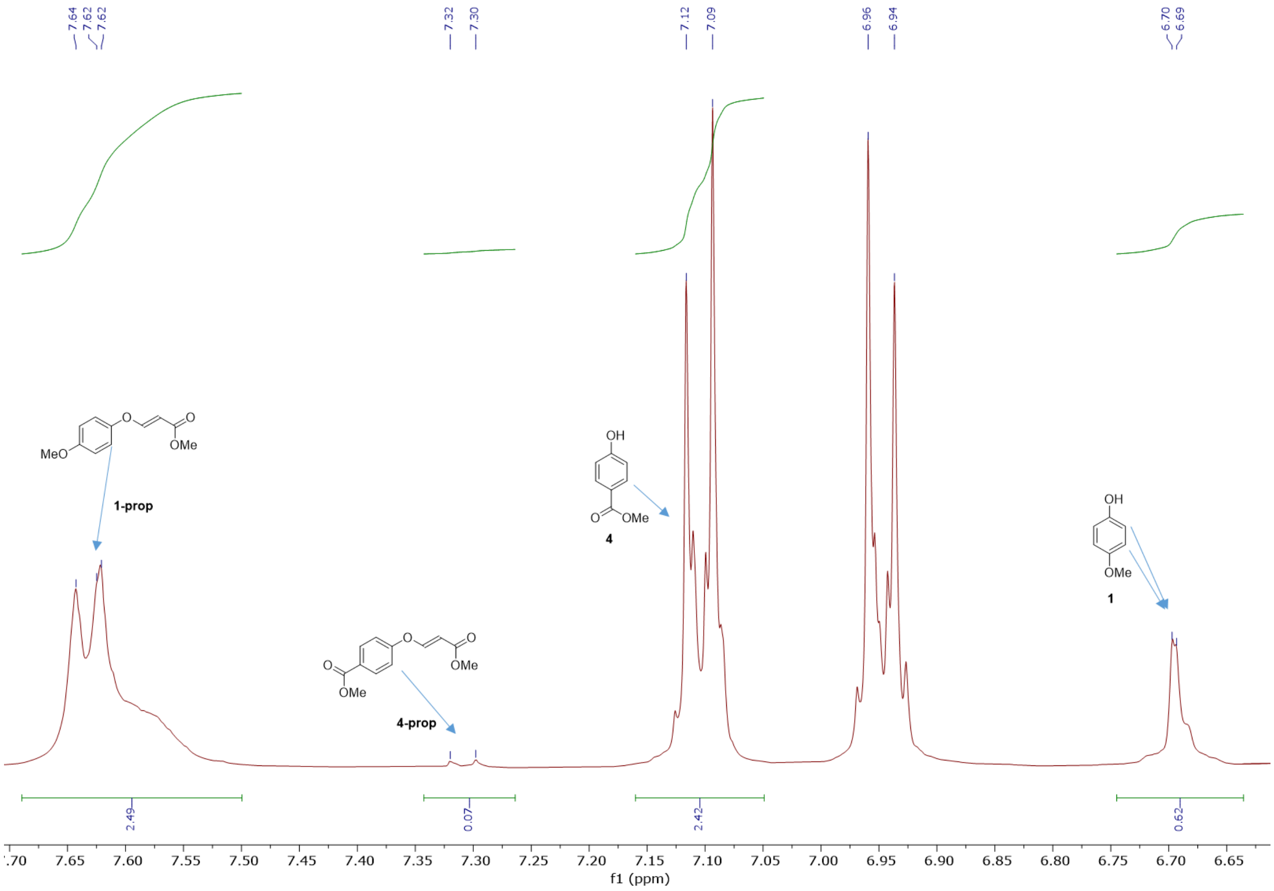

Compound	ppm	Area	Relative area[a]	Molar Fraction
1	6.70	0.62	0.16	0.06
4-propEt	7.31	0.07	0.035	0.01
4	7.11	2.42	1.21	0.46
1-propEt	7.62	2.49	1.245	0.47

[a]Relative area is calculated by dividing the total area by the number of equivalent hydrogens.

$$K = \frac{[0.46][0.47]}{[0.06][0.01]} = 360.33$$

System 4

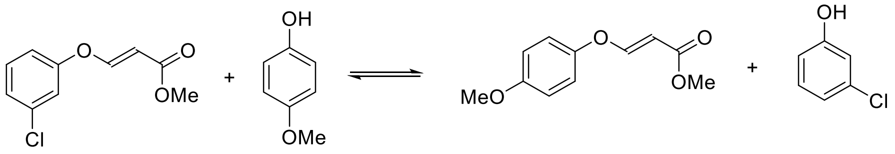

5-prop **1** **1-prop** **5**

$$K = \frac{[5][1 - \mathrm{prop}]}{[1][5 - \mathrm{prop}]}$$

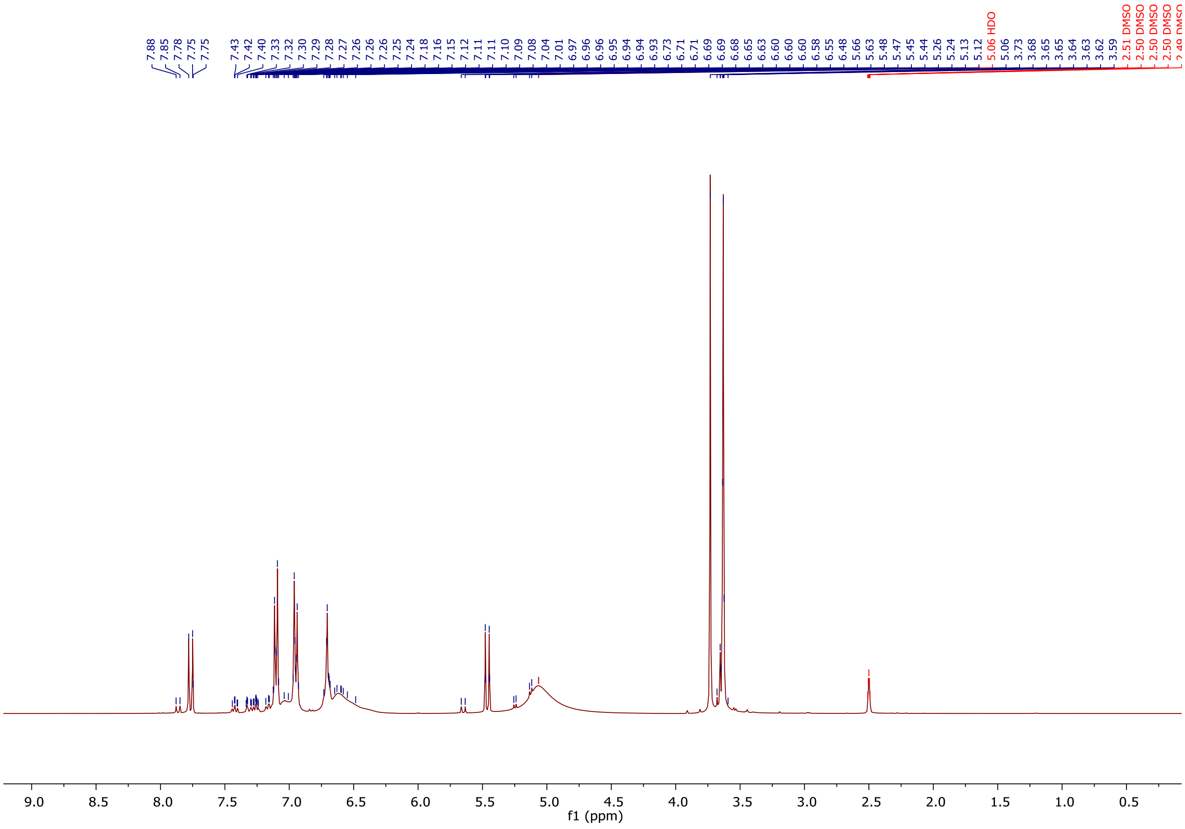

DMSO-d_6 (400 MHz)

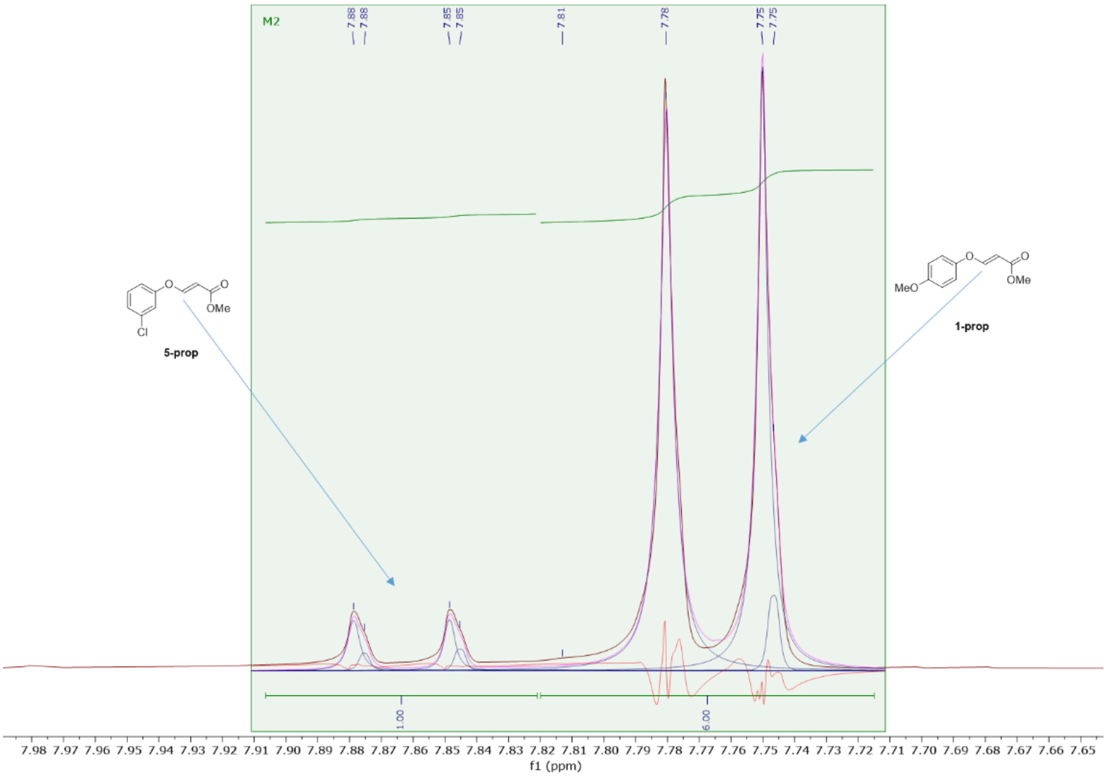

Due to overlappings in the NMR spectrum, we took the ratio between **1-prop** and **5-prop** and we assumed the ratio between **1** and **5** was exactly the inverse.

Compound	ppm	Area	Molar Fraction
1	-	1.00	0.07
5-propEt	7.86	1.00	0.07
5	-	6.00	0.43
1-propEt	7.65	6.00	0.43

$$K = \frac{[0.43][0.43]}{[0.07][0.07]} = 37.73$$

System 5

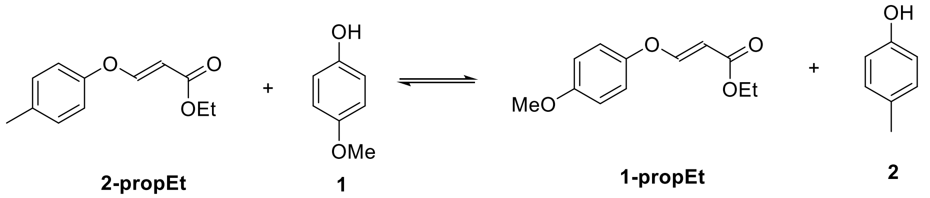

$$K = \frac{[2][1 - \text{propEt}]}{[1][2 - \text{propEt}]}$$

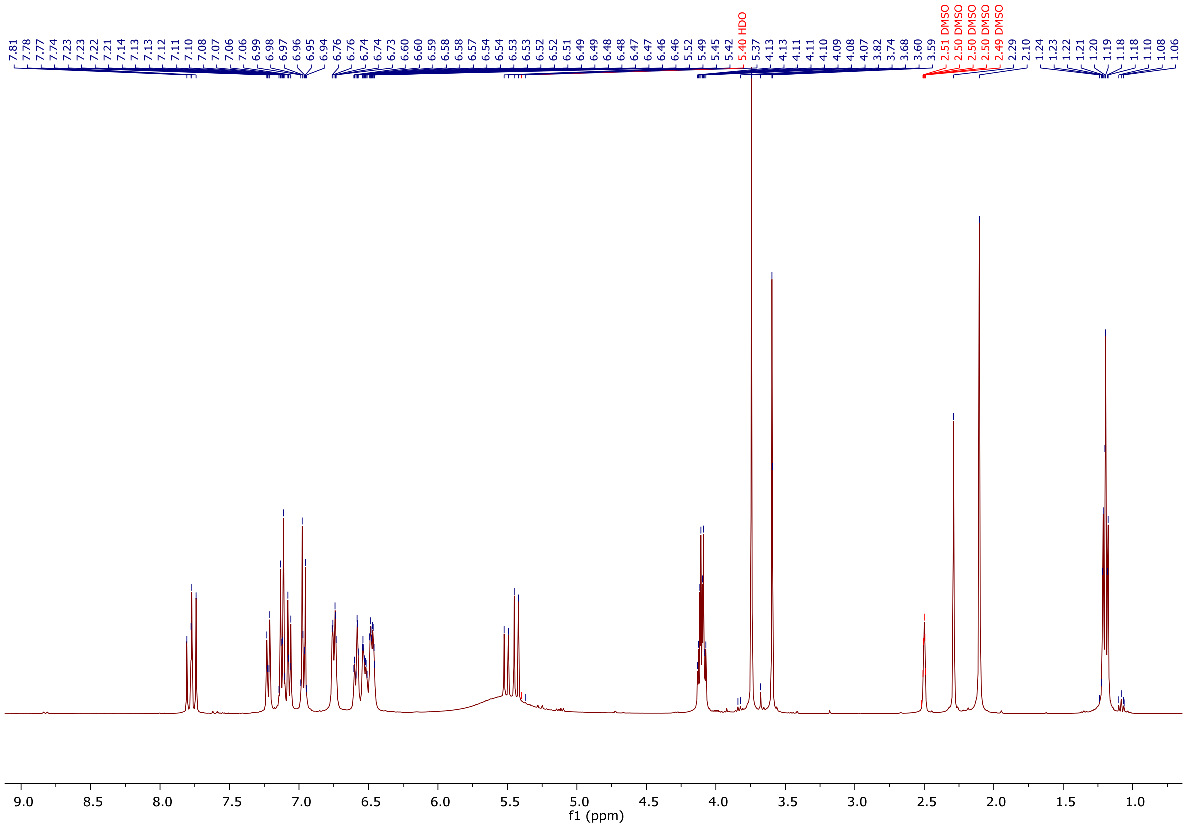

DMSO-d_6 (400 MHz)

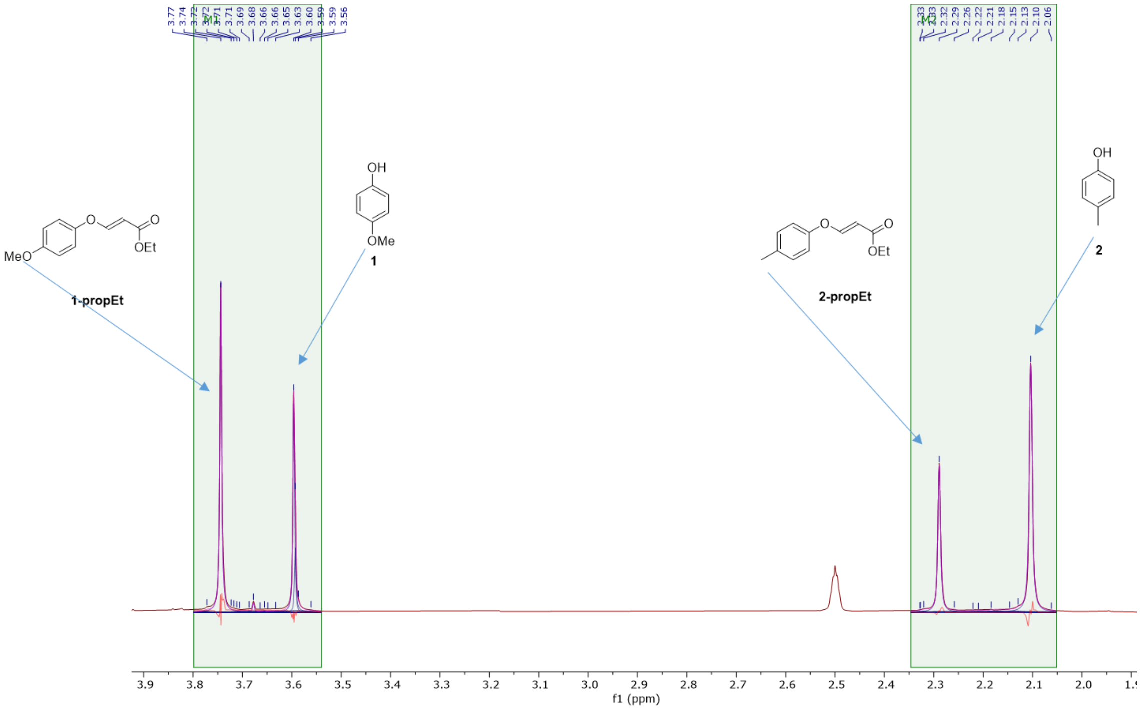

Compound	ppm	Area	Molar Fraction
1	3.60	230113818.5	0.17
2-propEt	2.29	177187226.0	0.19
2	2.10	356766972.01	0.35
1-propEt	3.74	258321008.3	0.29

$$K = \frac{[0.35][0.29]}{[0.17][0.19]} = 3.14$$

System 6

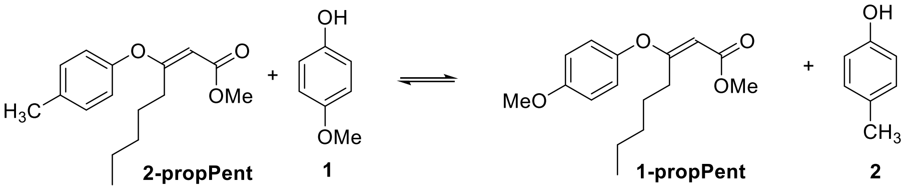

$$K = \frac{[2][1 - \text{propPent}]}{[1][2 - \text{propPent}]}$$

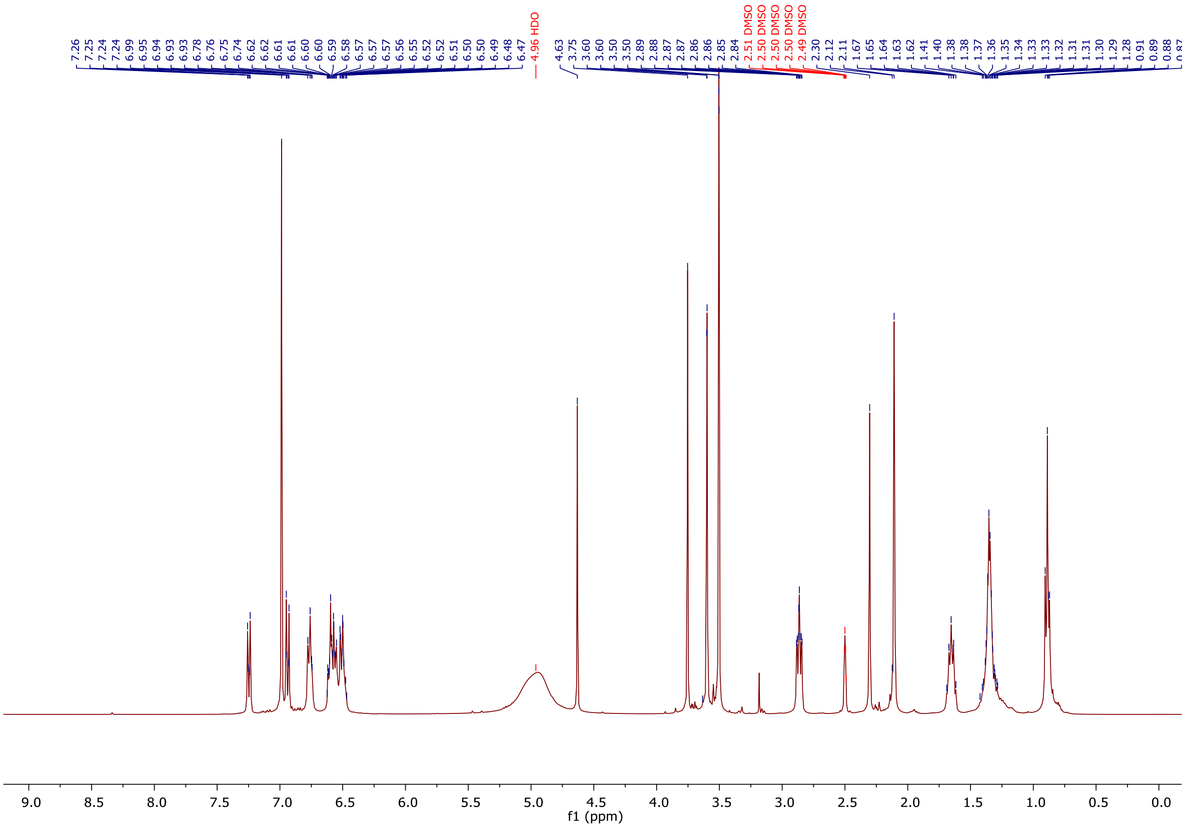

DMSO-d_6 (400 MHz)

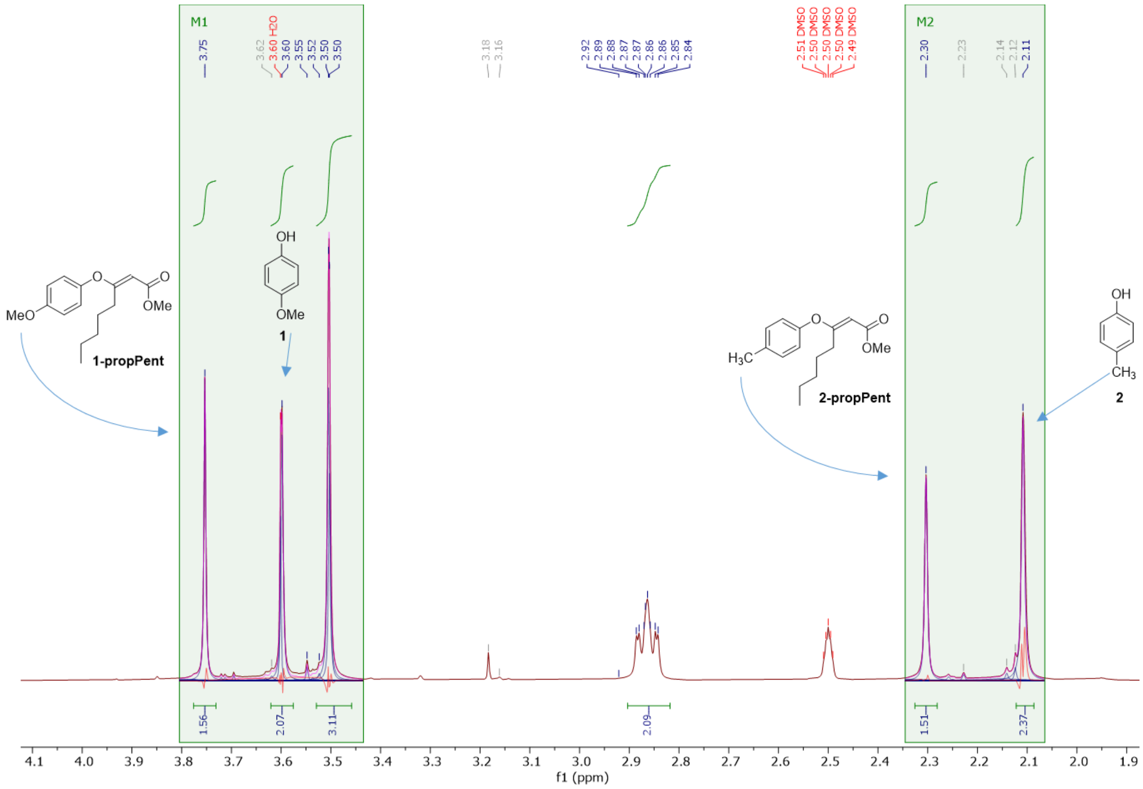

Compound	ppm	Area	Molar Fraction
1	3.60	183205645.94	0.22
2-propPent	2.30	181879833.0	0.22
2	2.11	274267346.65	0.33
1-propPent	3.75	184190325.24	0.23

$$K = \frac{[0.33][0.23]}{[0.22][0.22]} = 1.57$$

System 7

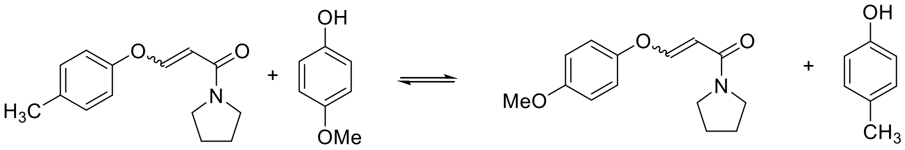

2-propNsec **1** **1-propNsec** **2**

$$K = \frac{[2][1 - \text{propNsec}]}{[1][2 - \text{propNsec}]}$$

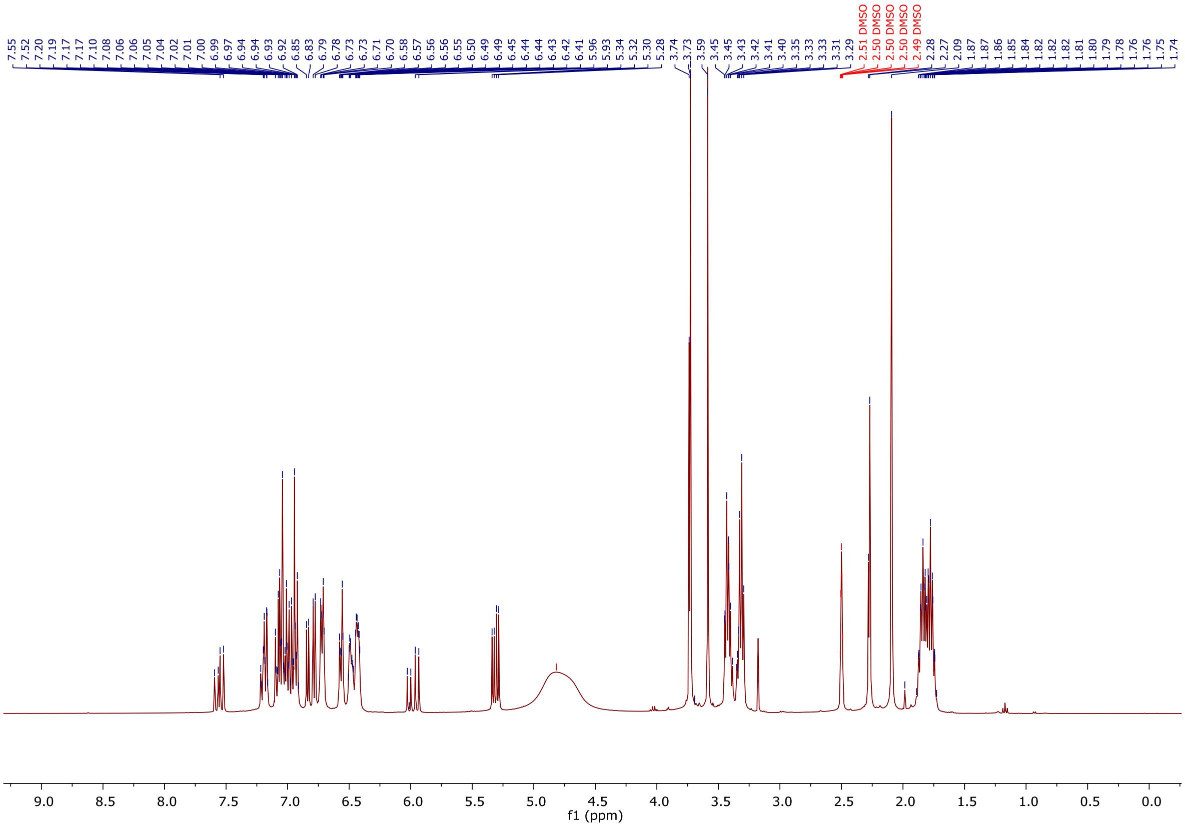

DMSO-d_6 (400 MHz)

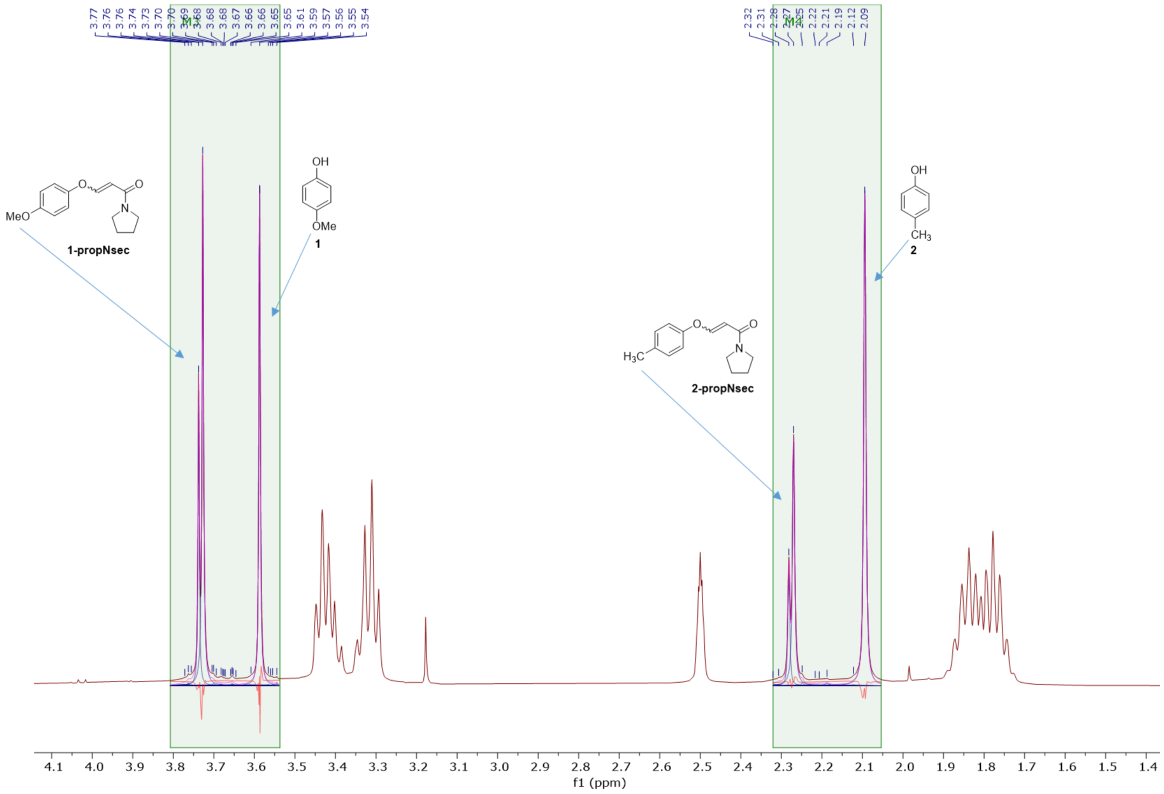

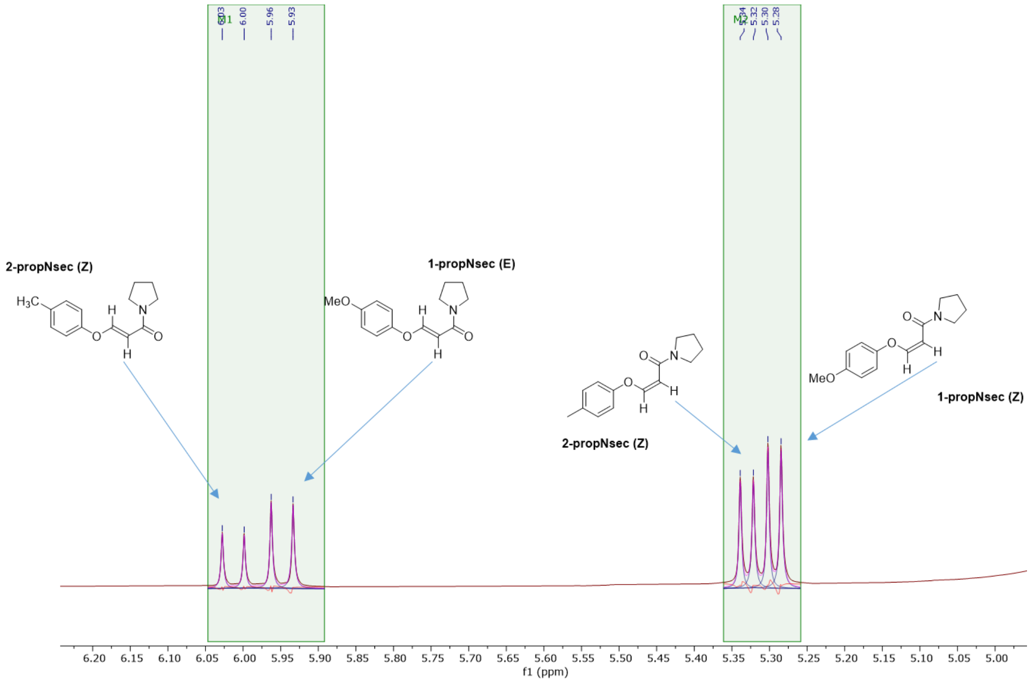

Compound	ppm	Area	Total Area	Molar Fraction
1	3.59	230113818.5	2210077.35	0.19
2-propNsec (Z)	2.27	177187226.0	246047154.23	0.20
2-propNsec (E)	2.28	68859928.23		
2	2.09	356766972.01	1982317.78	0.30
1-propNsec (Z)	3.73	258321008.3	376982733.7	0.31
1-propNsec (E)	3.74	118661725.4		

$$K = \frac{[0.30][0.31]}{[0.19][0.20]} = 2.45$$

Equilibria in CD_3CN

To a solution of **1** (0.081 mmol, 1 equiv.), and **2-Prop** (0.081 mmol, 1 equiv.) in CD_3CN as a deuterated solvent (0.5 mL) was added Cs_2CO_3 (0.163 mmol, 2 equiv.). The reaction mixture was monitored by ^1H NMR at 25°C until thermodynamic equilibrium was reached in 4h.

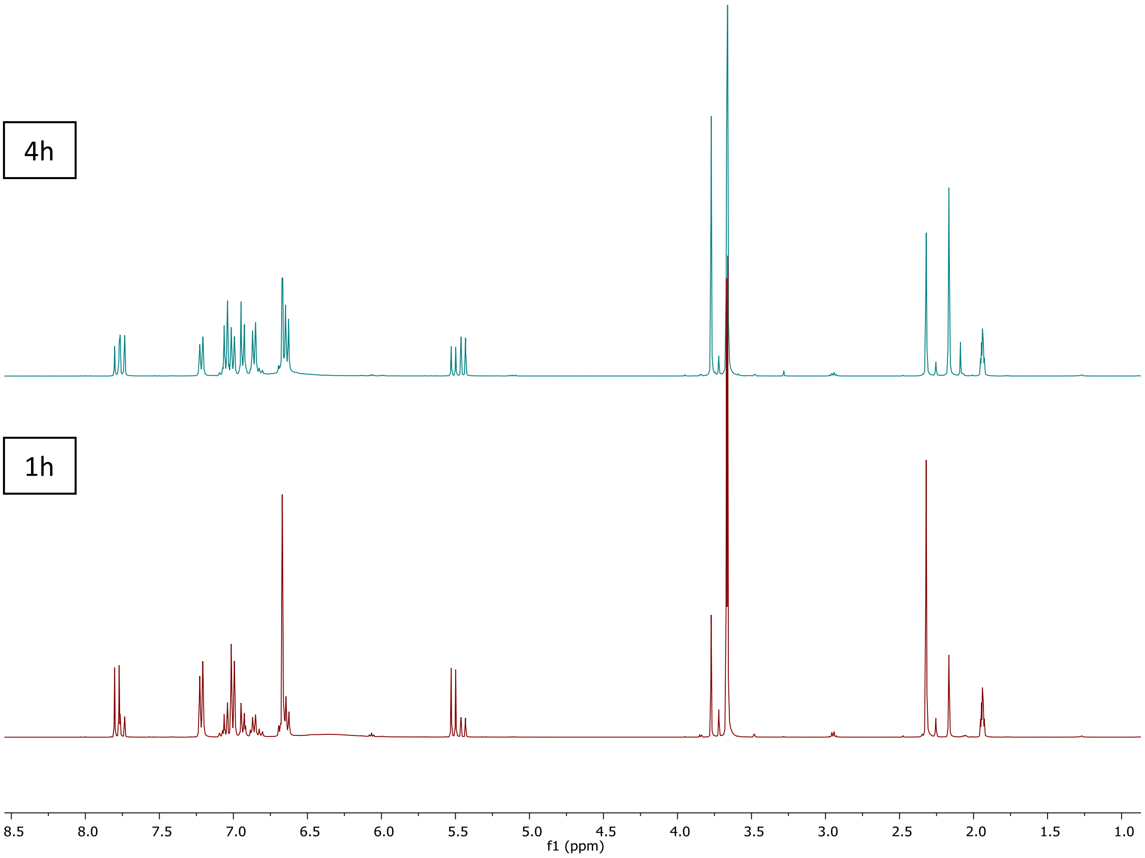

CD_3CN (400 MHz)

The reaction mixture was left for 45 days, and no major decomposition was observed.

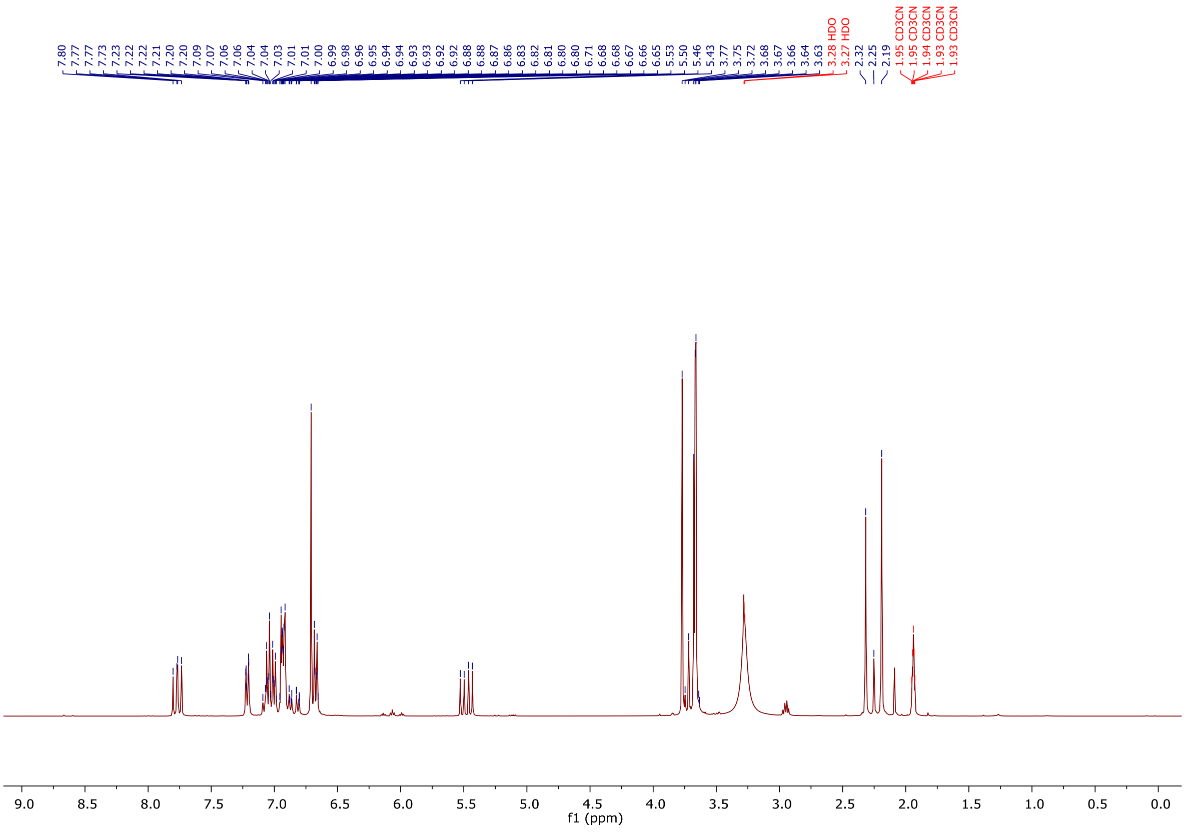

CD3CN (400 MHz)

Kinetic Studies

General procedure

To a solution of the corresponding vinyl ether derivative (0.20 mmol, 1 equiv.) and phenol (0.20 mmol, 1 equiv.) in DMSO-d_6 as a deuterated solvent (0.6 mL) was added the base DMAP or Cs_2CO_3 (0.40 mmol, 2 equiv.). The reaction mixture was monitored by ^{1}H NMR at 90°C in case of using DMAP and at 25ºC in case of using Cs_2CO_3.

*Reverse reaction with DMAP: p-cresol (**2**) with vinyl ether **1prop***

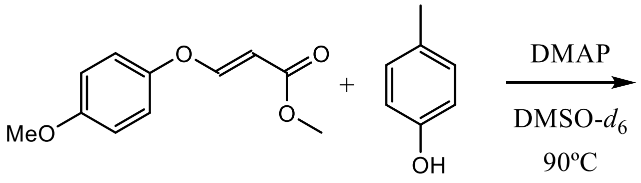

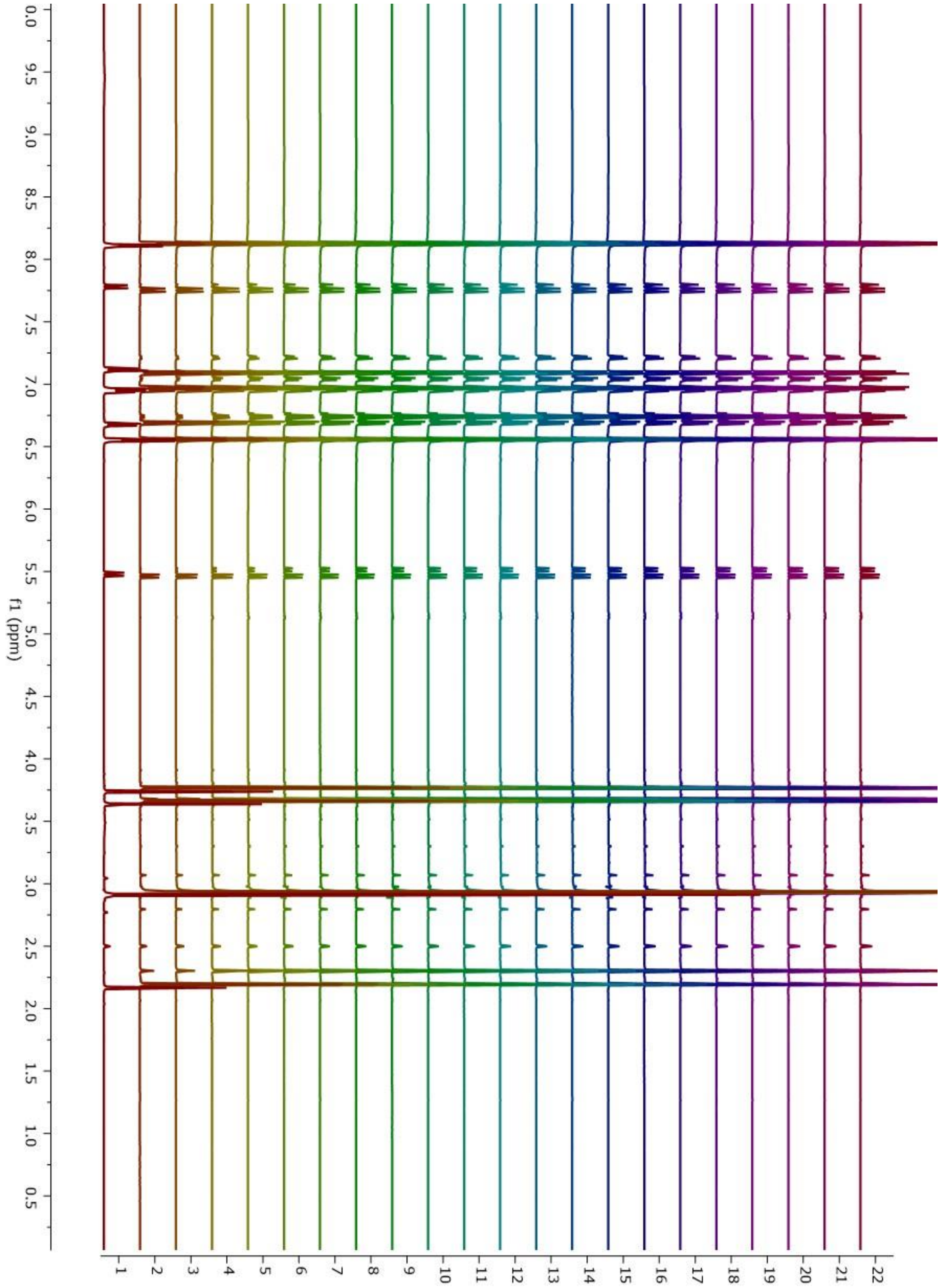

Figure S1. ^{1}H NMR sequence for the exchange equilibrium between **1-prop** and p-cresol (**2**) in DMSO-d_6 at 90°C.

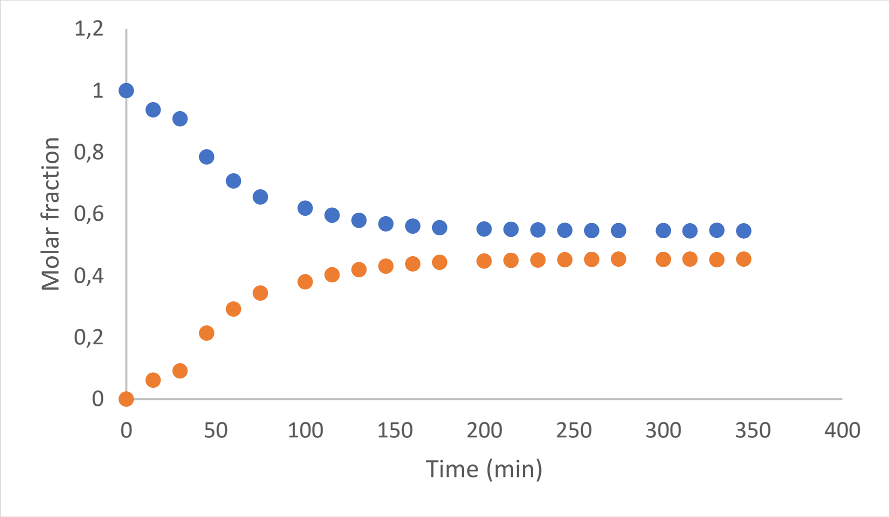

Figure S2. Kinetics of the reverse reaction, between **1-prop** and p-cresol (**2**) with DMAP in DMSO-d_6 at 90°C. Orange dots represent the molar fraction of **2-prop**, while blue dots represent the molar fraction of free p-cresol (**2**). The molar fraction was calculated taking into account only **2-prop** and **2**.

*Forward reaction with DMAP: **2-prop** with p-methoxy phenol (**1**)*

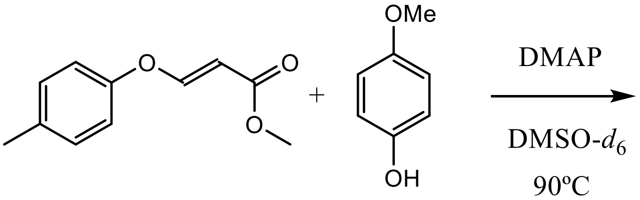

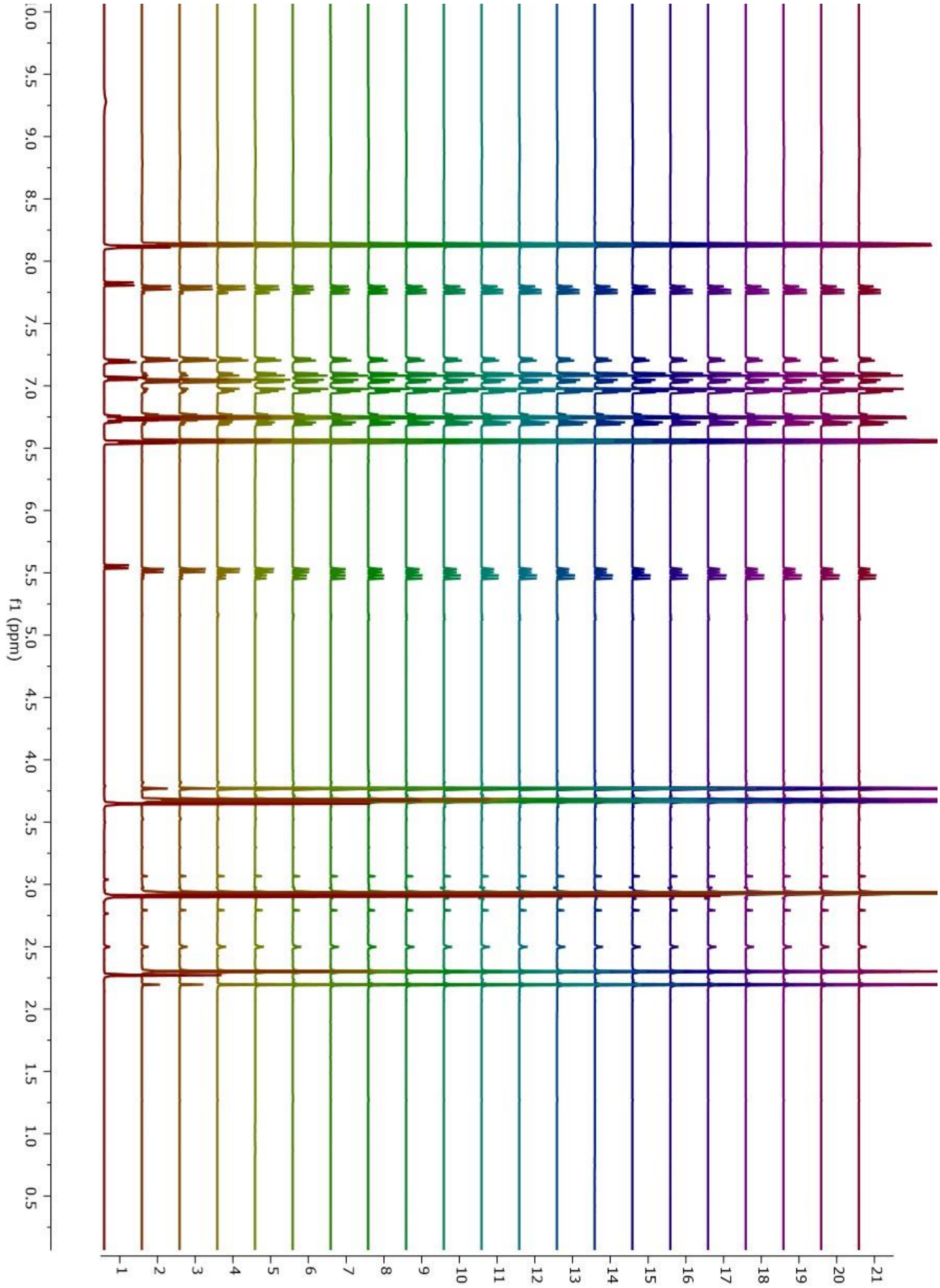

Figure S3. ^{1}H NMR sequence for the exchange equilibrium between **2-prop** and p-methoxy phenol (**1**) in DMSO-d_6 at 90°C.

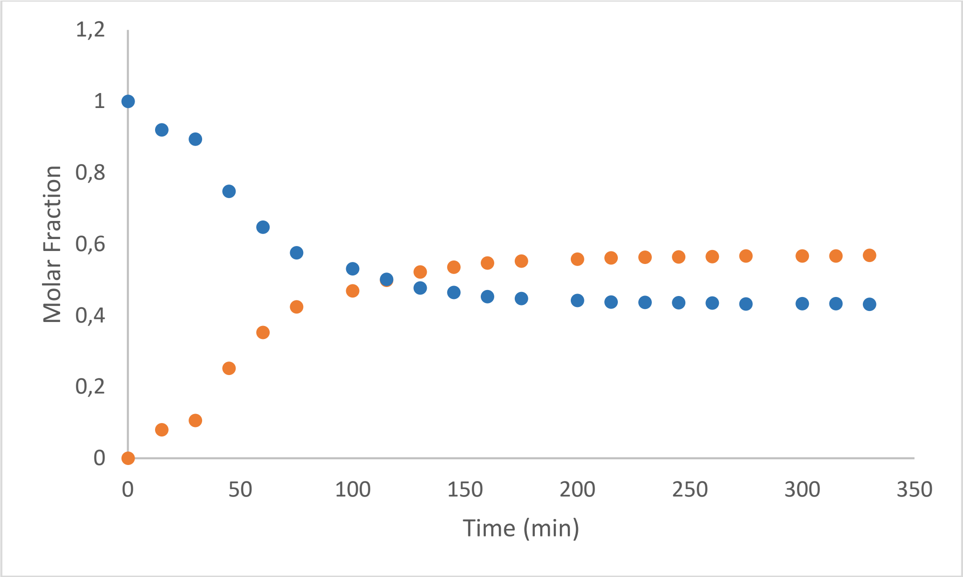

Figure S4. Kinetics of the forward reaction between **2-prop** and p-methoxy phenol (**1**) with DMAP in DMSO-d6 at 90°C. Orange dots represent the molar fraction of p-cresol attached (**1-prop**), while blue dots represent the molar fraction of free p-cresol (**1**). The molar fraction was calculated taking into account only **1-prop** and **1**.

*Forward reaction with Cs_2CO_3: **2-prop** with p-methoxy phenol (**1**)*

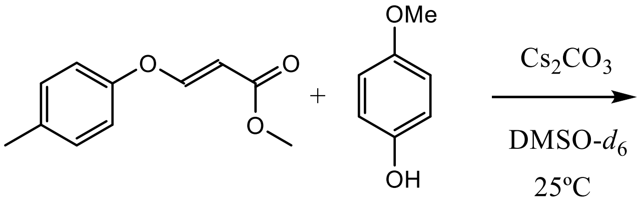

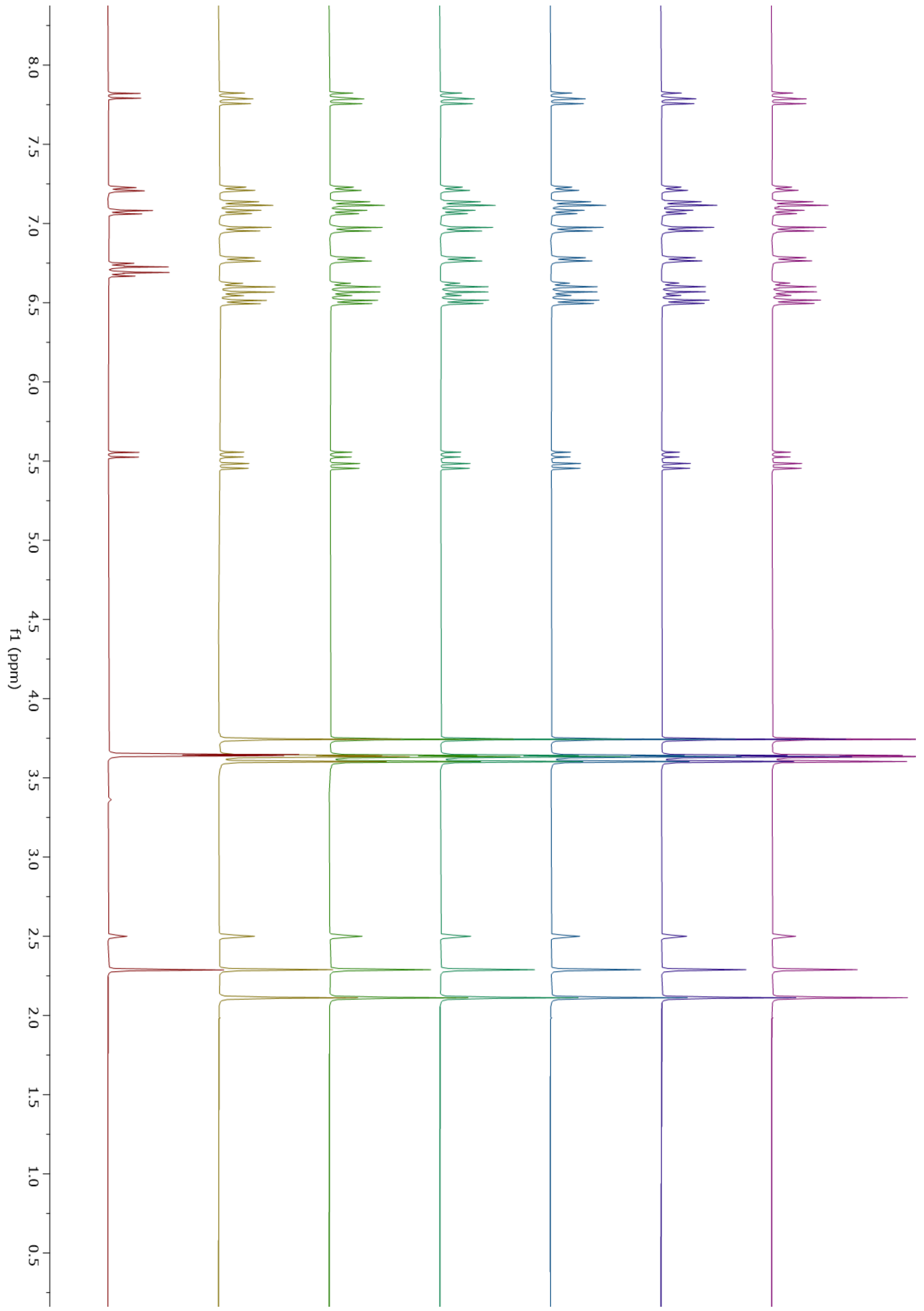

Figure S5. ^{1}H NMR sequence for the exchange equilibrium between **2-prop** and p-methoxy phenol (**1**) with Cs_2CO_3 in DMSO-d_6 at 25°C.

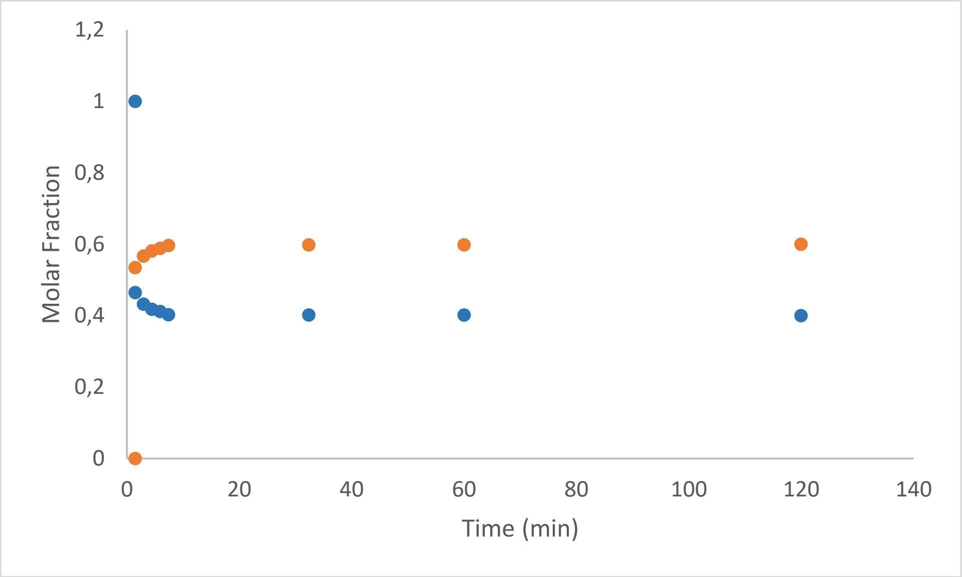

Figure S6. Kinetics of the forward reaction between **2-prop** and p-methoxy phenol (**1**) with Cs_2CO_3 in DMSO-d_6 at 25°C. Orange dots represent the molar fraction of p-cresol attached (**1-prop**), while blue dots represent the molar fraction of free p-cresol (**1**). The molar fraction was calculated taking into account only **1-prop** and **1**.

Reaction with ketones and primary amides.

We carried out exchange reactions with phenyl ethynyl ketone (keto) and with the propiolamide of the p-anisidine. In both cases, although reactions seem to reach equilibrium, ^{1}H NMR of the crude reaction mixtures over time shows decomposition of the vinyl ethers initially formed. Therefore a proper quantification is not possible.

To a solution of p-methoxyphenol (**1**) and p-cresol (**2**) (0.0081 mmol of each one, 1 equiv.), and the corresponding activated alkyne (0.0081 mmol, 1 equiv.) in DMSO-d_6 as a deuterated solvent (0.5 mL) was added Cs_2CO_3 (0.0163 mmol, 2 equiv.). The reaction mixture was monitored by 1H NMR at 25°C.Compatibility of the Hydroxyl-yne reaction with the imine exchange

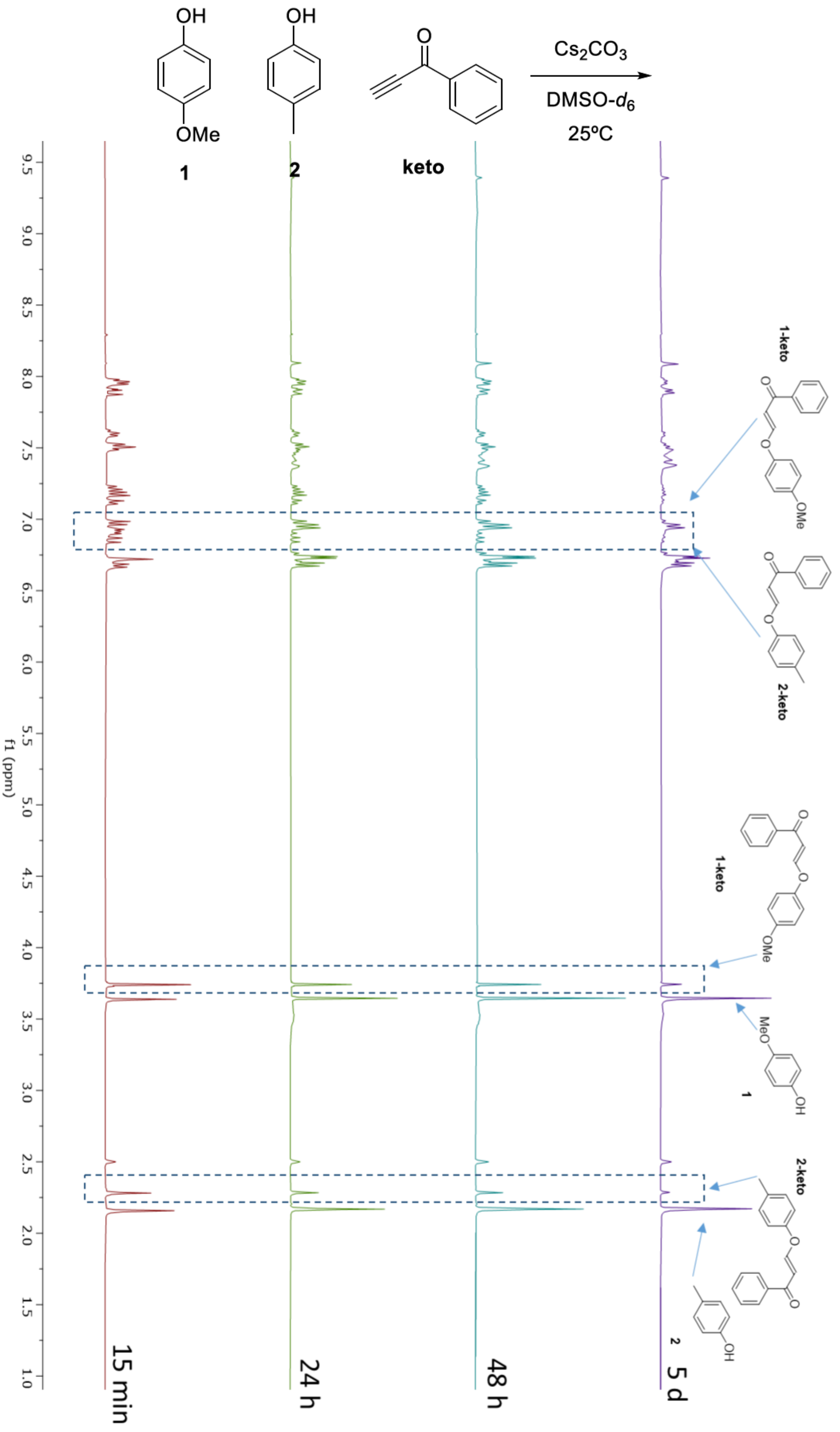

DMSO-d_6 (400 MHz)

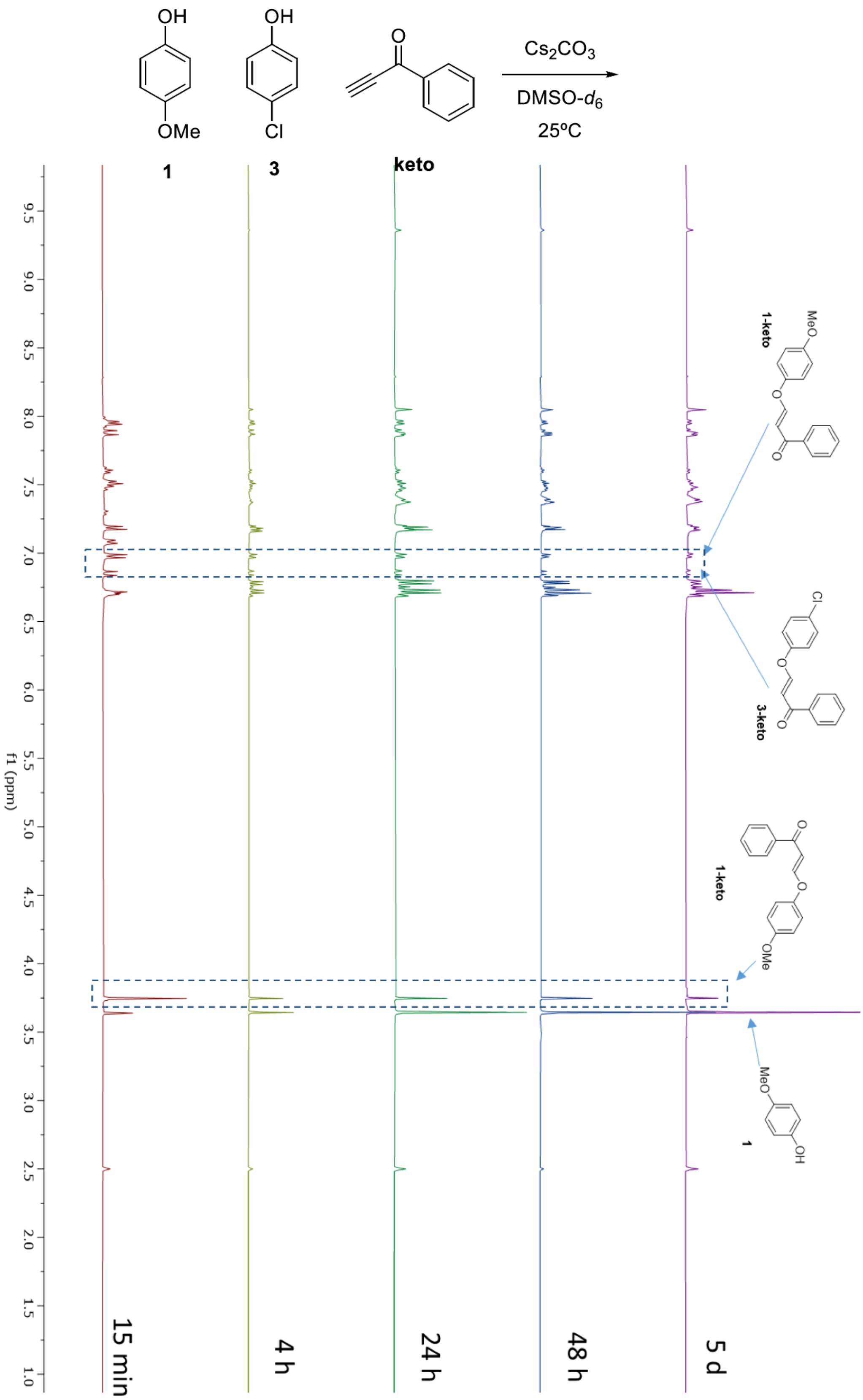

DMSO-d_6 (400 MHz)

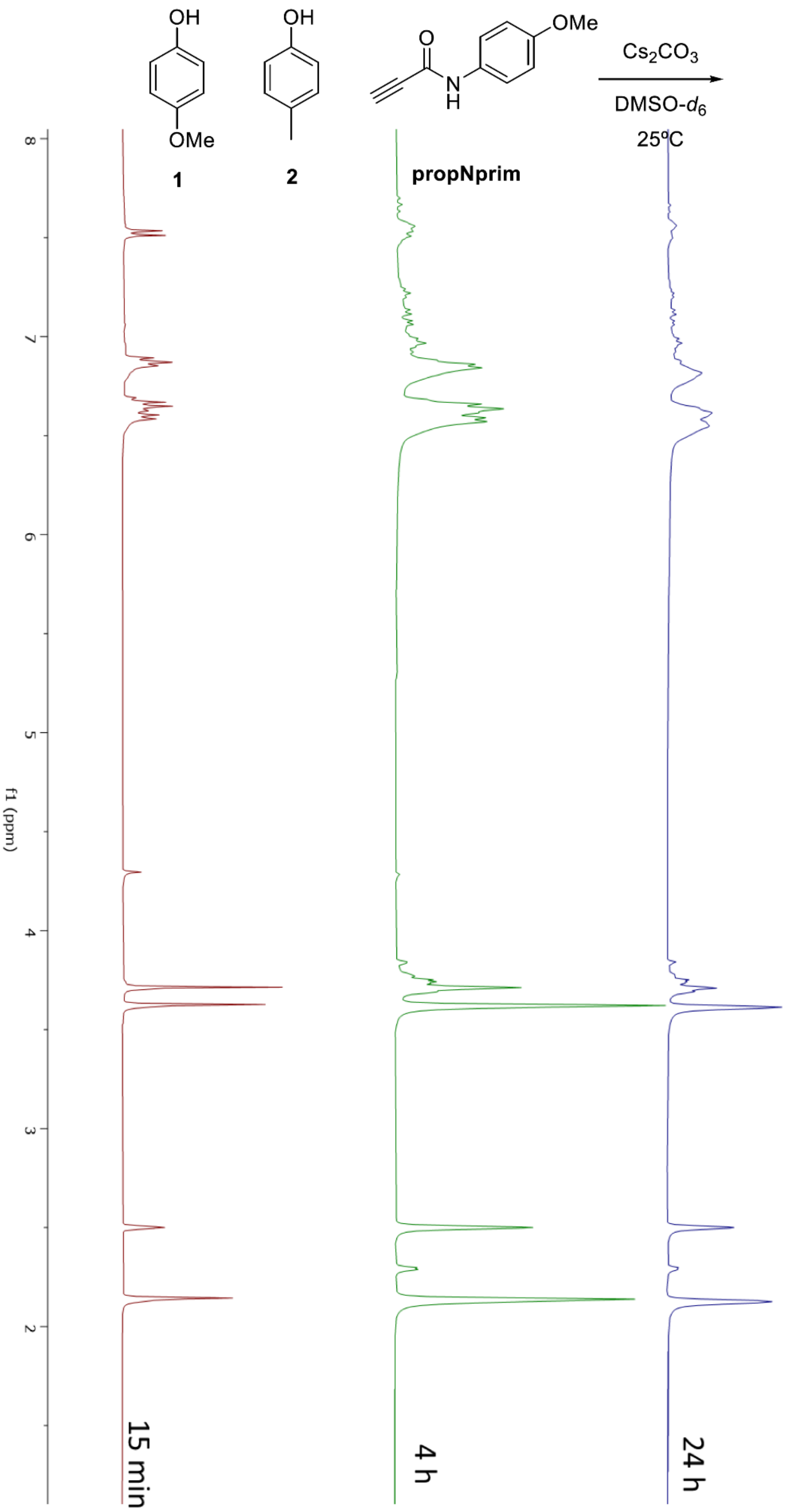

DMSO-d_6 (400 MHz)

Attempts of dynamic hydroxyl-yne with alkyl alcohol.

Several experiments were carried out to check if alkyl alcohols can undergo dynamic hydroxyl-yne reaction. None of the conditions we tested was successful, only unreacted starting materials.

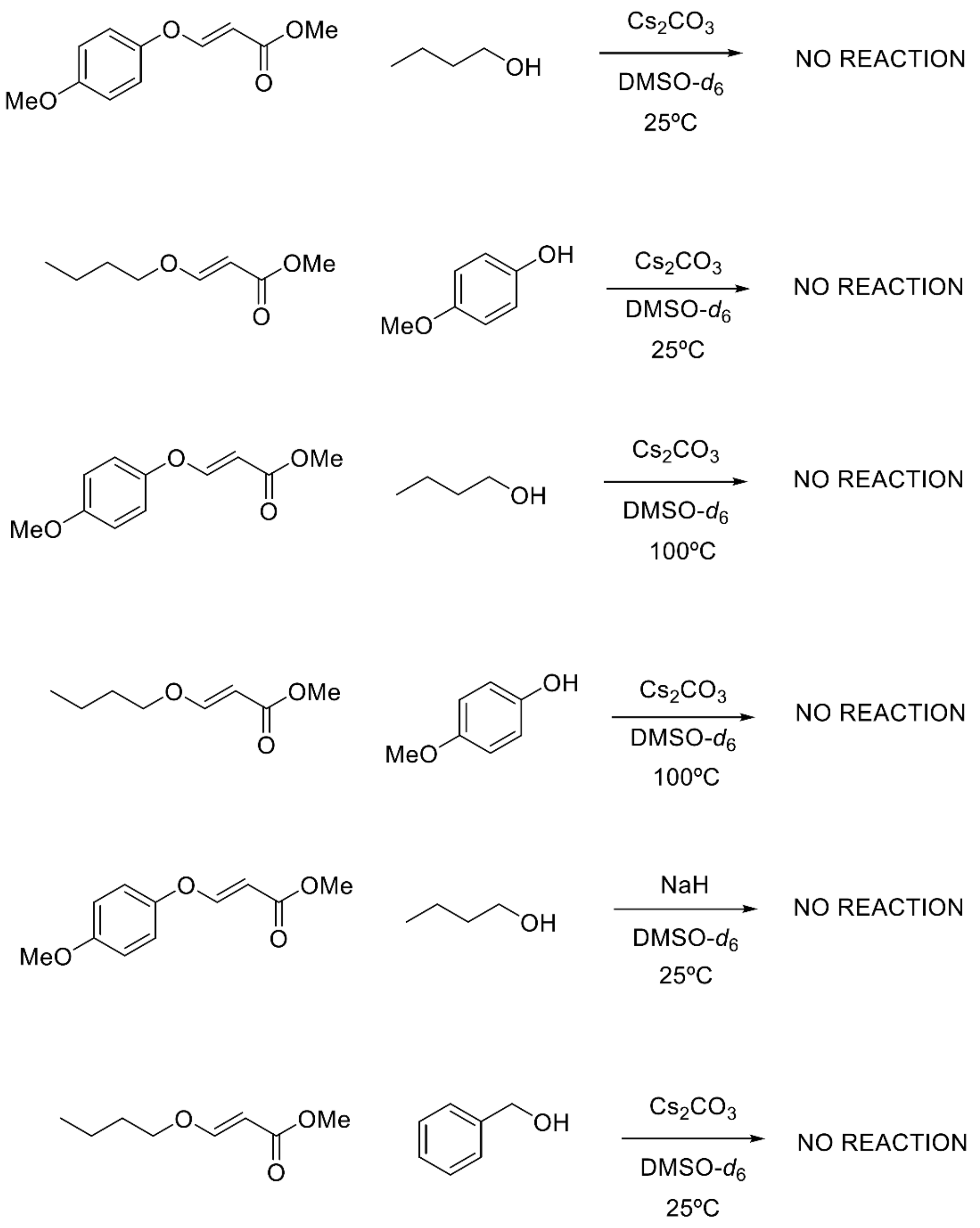

Exchange was not successful even with commercial sodium ethoxide with **2-prop** in different solvents (DMSO, MeOH, CH_3CN), not even heating the reaction mixture.

Compatibility of the hydroxyl-yne reaction with the imine exchange.

4-aminophenol (16 mg, 0.147 mmol) and methyl propiolate (12.3 mg, 0.147 mmol) were solved in DMSO-*d*6 (0.8 ml). Then Cs_2CO_3 (96 mg, 0.294 mmol) was added. After 60 minutes, reaction was clearly complete, obtaining the vinyl ether with the amino group unreacted. Then 4-bromobenzaldehyde (27.20 mg, 0.147 mmol) and Na_2SO_4 were added. The imine was slowly formed and the equilibrium almost completely shifted towards the imine after 7 days.

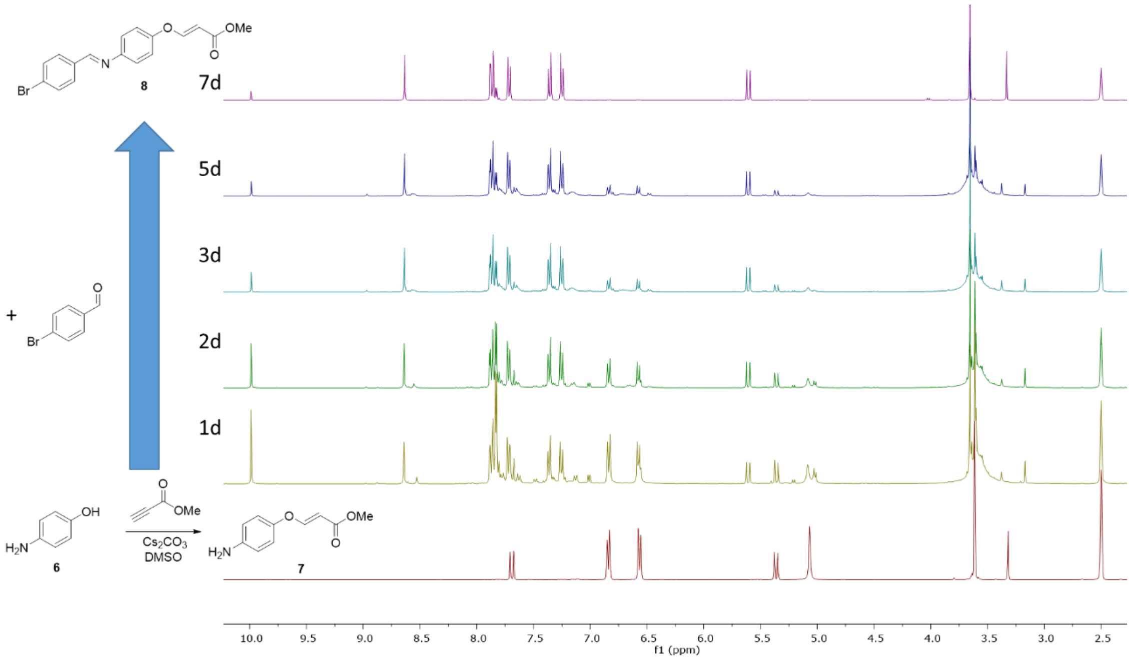

DMSO-*d*6 (400 MHz)

Synthesis of hemicryptophane (±)11 and disassembly studies.

CTV derivative **(±)9** was synthesized as previously reported.[1]

Tripropiolate ester **10** was synthesized by adding propiolic acid (105 mg, 1.5 mmol) to a mixture of 2,4,6-triethyl-1,3,5-tribromomethyl benzene (200 mg, 0.45 mmol) in acetonitrile (5 ml), and finally K_2CO_3 (270 mg, 1.95 mmol) was added. The reaction mixture was heated at 45°C with a Heat-on™ block for 12h. Then, reaction mixture was cooled down to room temperature, water was added and it was extracted with ethyl acetate (3 x 10 ml). The organic layers were dried with Na_2SO_4, filtered and the solvent removed. The crude was purified by chromatographic column yielding 154 mg of **10** (83% yield).

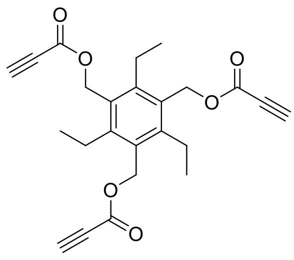

10

(2,4,6-triethylbenzene-1,3,5-triyl)tris(methylene) tripropiolate (10). Purified by silica gel chromatography (EtOAc/Hexane: 20:80 v /v). white solid (154mg, 83%).

^{1}H NMR (400 MHz, $CDCl_3$) δ 5.34 (s, 6H), 2.89 (s, 3H), 2.78 (q, *J* = 7.6 Hz, 6H), 1.21 (t, *J* = 7.6 Hz, 9H).

^{13}C NMR (101 MHz, $CDCl_3$) δ δ 152.8, 147.7, 129.21, 75.5, 74.5, 62.4, 23.3, 16.4.

HR-MS (ESI$^+$, m/z): $[M+Na]^+$= $C_{24}H_{24}O_6Na$, calcd.: 431.1471; found 431.1477.

Hemicryptophane (±)11 was synthesized by solving the CTV derivative **(±)9** (41 mg, 0.1 mmol) and tripropiolate ester **10** (41 mg, 0.1 mmol) in DMSO (10 ml) and Cs_2CO_3 (196 mg, 0.6 mmol) was added. After 12h, ethyl acetate (10 ml) and water (10 ml) were added. The organic phase was collected and the aqueous phase was extracted with ethyl acetate (10 ml x 3 times). The organic layers were dried with Na_2SO_4, filtered and the solvent removed. The crude was purified by chromatographic column yielding 30 mg of cage **(±)11** (37% yield).

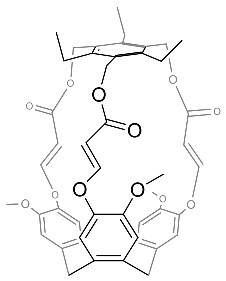

(±)11

^{1}H NMR (400 MHz, Acetonitrile-*d*$_3$) δ 7.22 (d, *J* = 12.2 Hz, 3H), 7.21 (s, 3H), 7.05 (s, 3H), 5.19 (d, *J* = 12.2 Hz, 3H), 5.06 (d, *J* = 12.4 Hz, 3H), 4.89 (d, *J* = 12.2 Hz, 3H), 4.75 (d, *J* = 13.7 Hz, 3H), 3.70 (s, 9H), 3.62 (d, *J* = 13.8 Hz, 3H), 2.56 (dp, *J* = 24.9, 7.4 Hz, 6H), 1.01 (t, *J* = 7.6 Hz, 9H).

^{13}C NMR (101 MHz, Acetonitrile-*d*$_3$) δ 167.1, 164.6, 150.5, 147.2, 144.0, 139. 8, 132. 9, 131.1, 124.0, 115.5, 99.6, 61.0, 56.7, 36.0, 23.3, 16.3.

HR-MS (ESI$^+$, m/z): $[M+Na]^+$= $C_{48}H_{48}O_{12}Na$, calcd.: 839.3043; found 839.3055.

For the disassembly study, cage **(±)11** (0.4 mg, 0.005 mmol) was solved in DMSO-d_6 (0.5 ml) and 10 equivalents of p-methoxyphenol **1** (6.2 mg, 0.05 mmol) were added, followed by 33 mg of Cs_2CO_3. Almost immediately the ^{1}H NMR showed a decomposition into several unknown fragments. Disassembly was achieved but not selective.

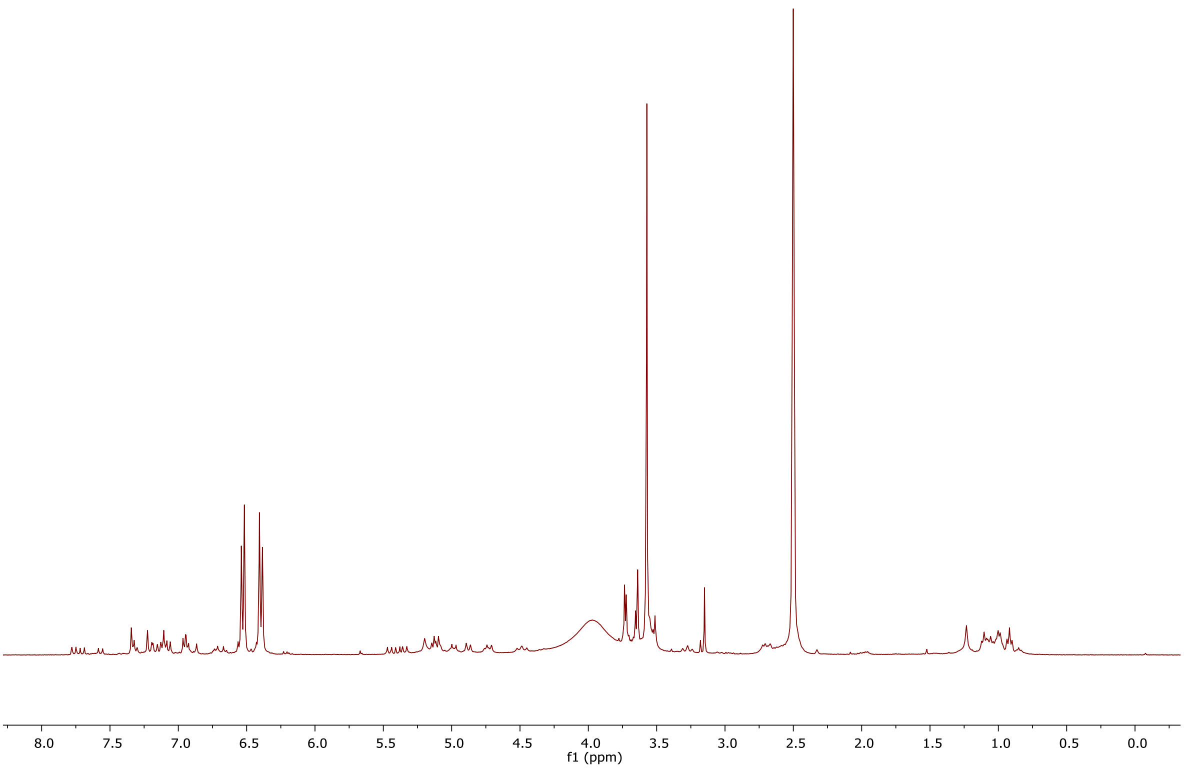

DMSO-d_6 (400 MHz)

NMR spectra

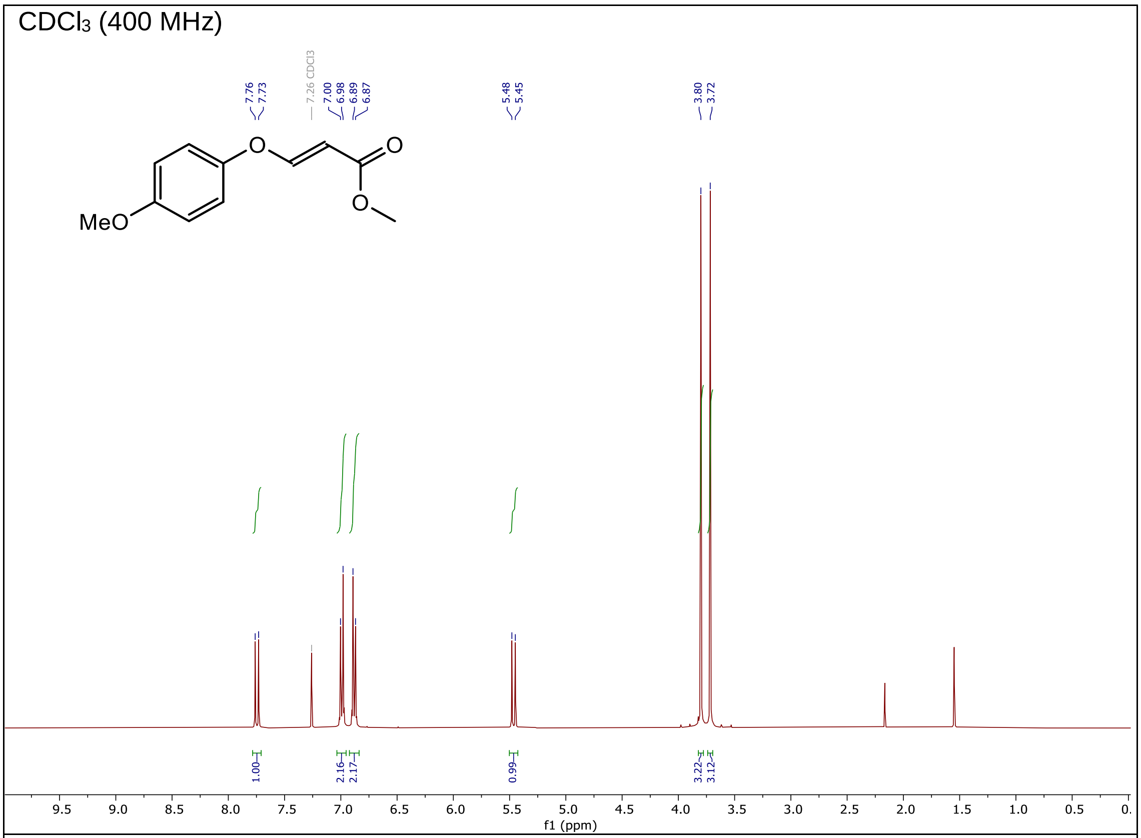
CDCl3 (400 MHz)
7.76
7.73
7.26 CDCl3
7.00
6.98
6.89
6.87
5.48
5.45
3.80
3.72
MeO
O
O
O
1.00
2.16
2.17
0.99
3.22
3.12
f1 (ppm)

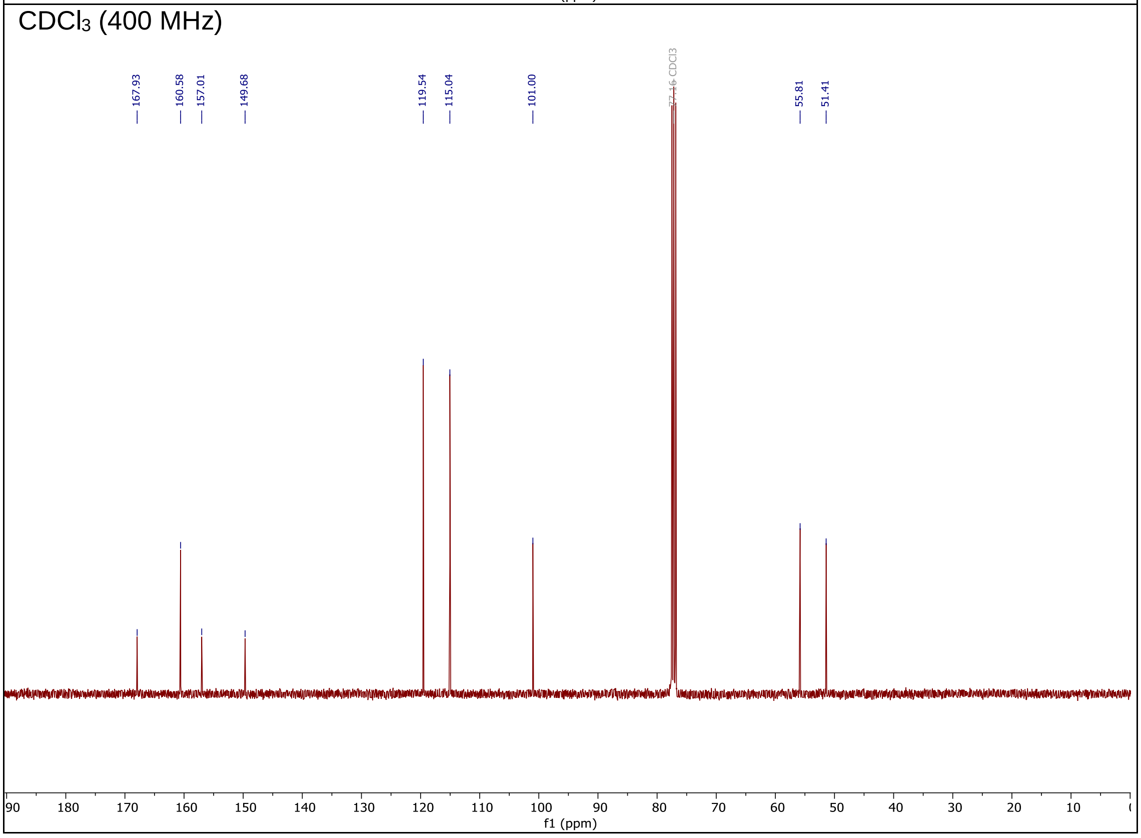
CDCl3 (400 MHz)
167.93
160.58
157.01
149.68
119.54
115.04
101.00
77.16 CDCl3
55.81
51.41
f1 (ppm)

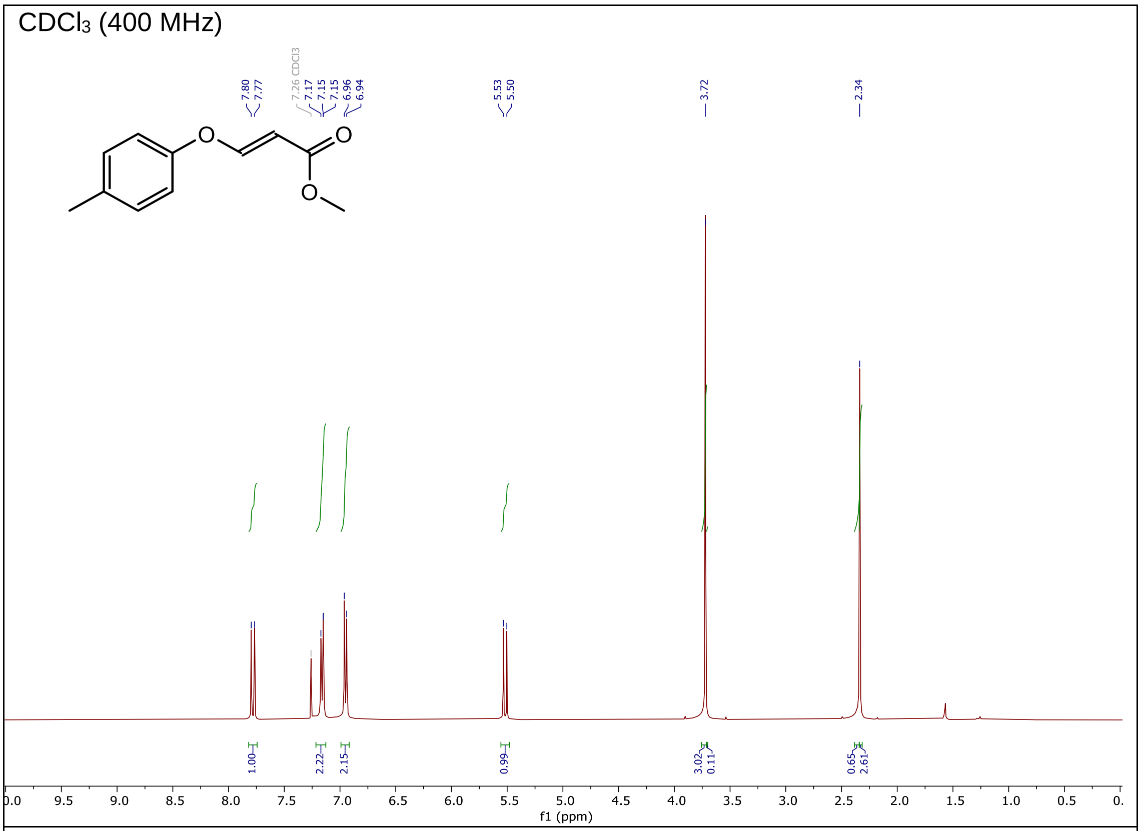
CDCl3 (400 MHz)
7.80
7.77
7.26 CDCl3
7.17
7.15
7.15
6.96
6.94
5.53
5.50
3.72
2.34
1.00
2.22
2.15
0.99
3.02
0.11
0.65
2.61
f1 (ppm)

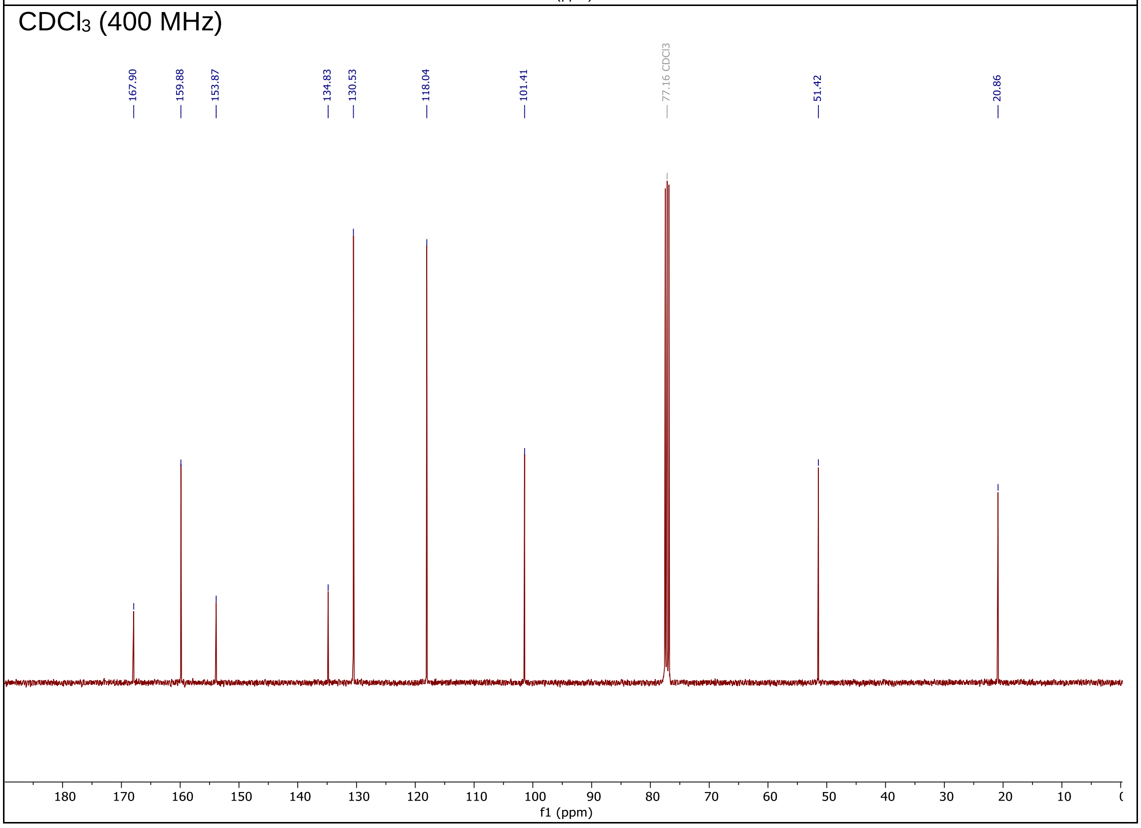
CDCl3 (400 MHz)
167.90
159.88
153.87
134.83
130.53
118.04
101.41
77.16 CDCl3
51.42
20.86
f1 (ppm)

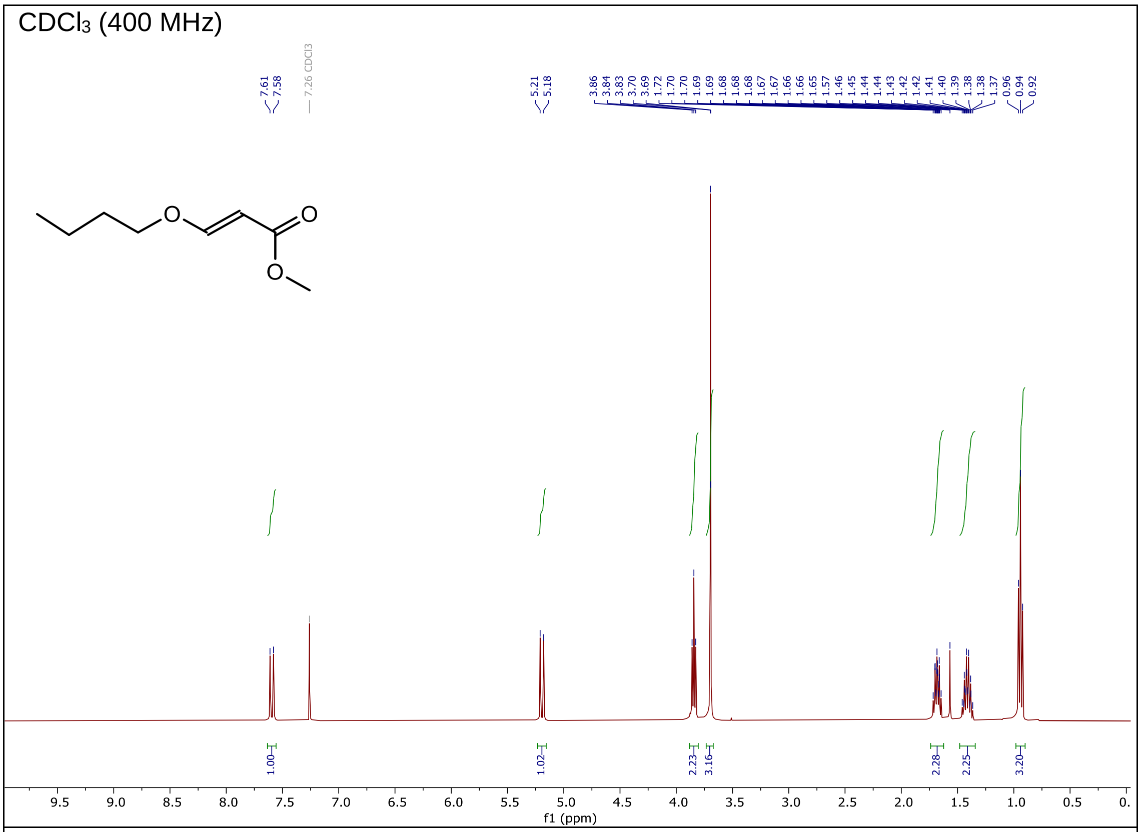
CDCl3 (400 MHz)
7.61
7.58
7.26 CDCl3
5.21
5.18
1.00
1.02
2.23
3.16
2.28
2.25
3.20
f1 (ppm)

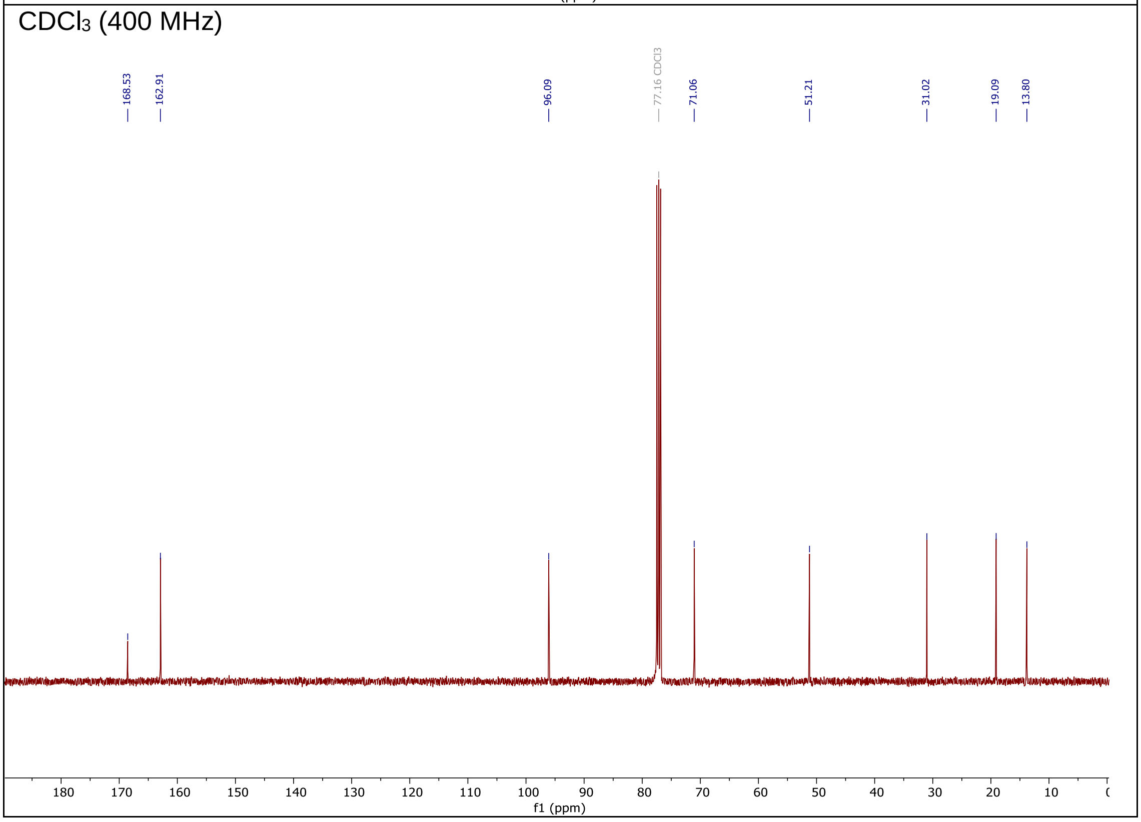
CDCl3 (400 MHz)
168.53
162.91
96.09
77.16 CDCl3
71.06
51.21
31.02
19.09
13.80
f1 (ppm)

CD₃CN (400 MHz)

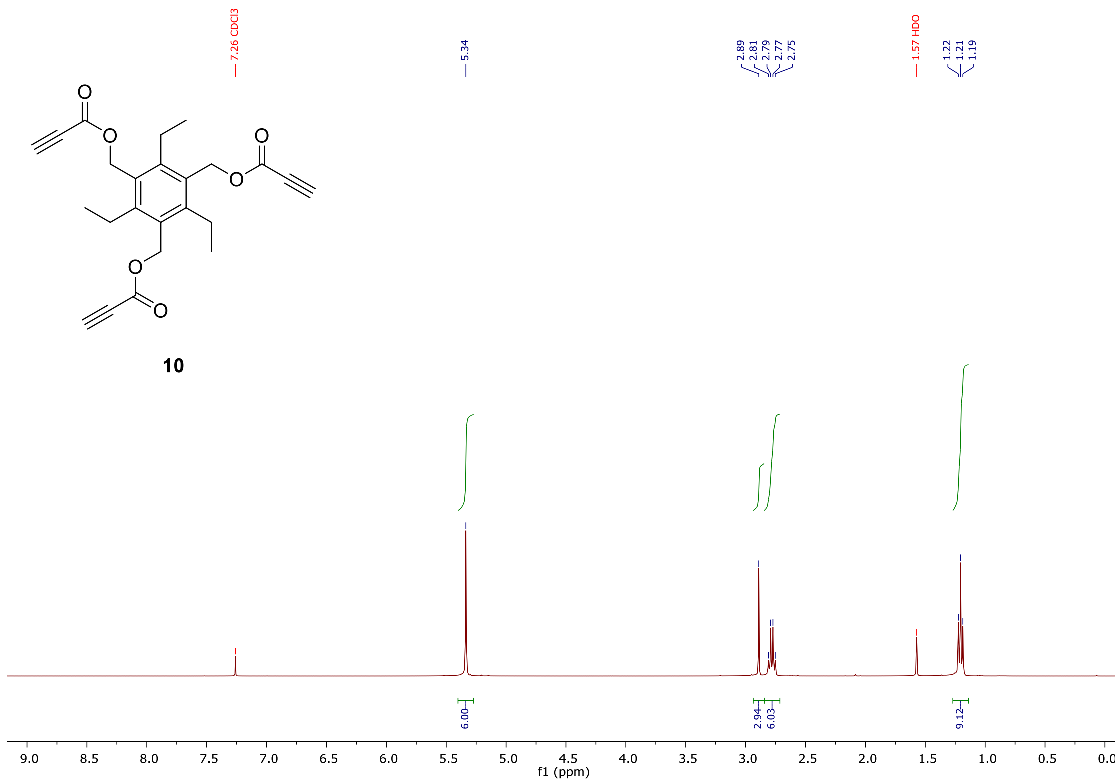

CD₃CN (400 MHz)

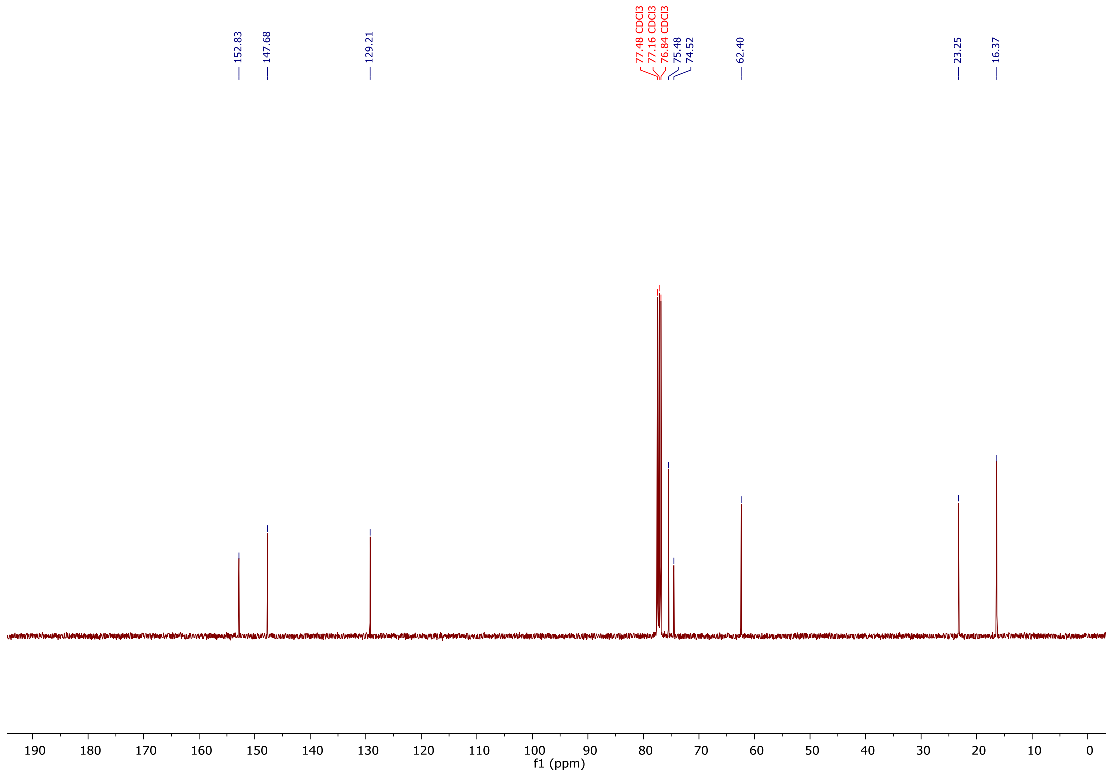

CD3CN (400 MHz)

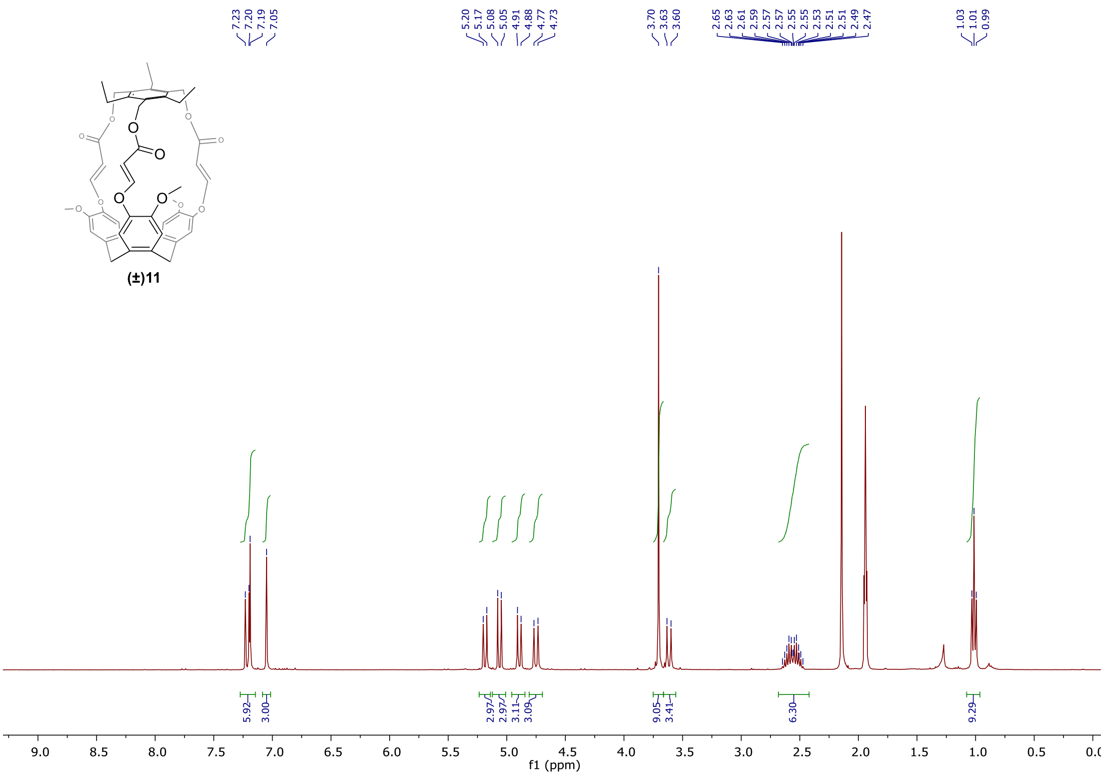

CD3CN (400 MHz)

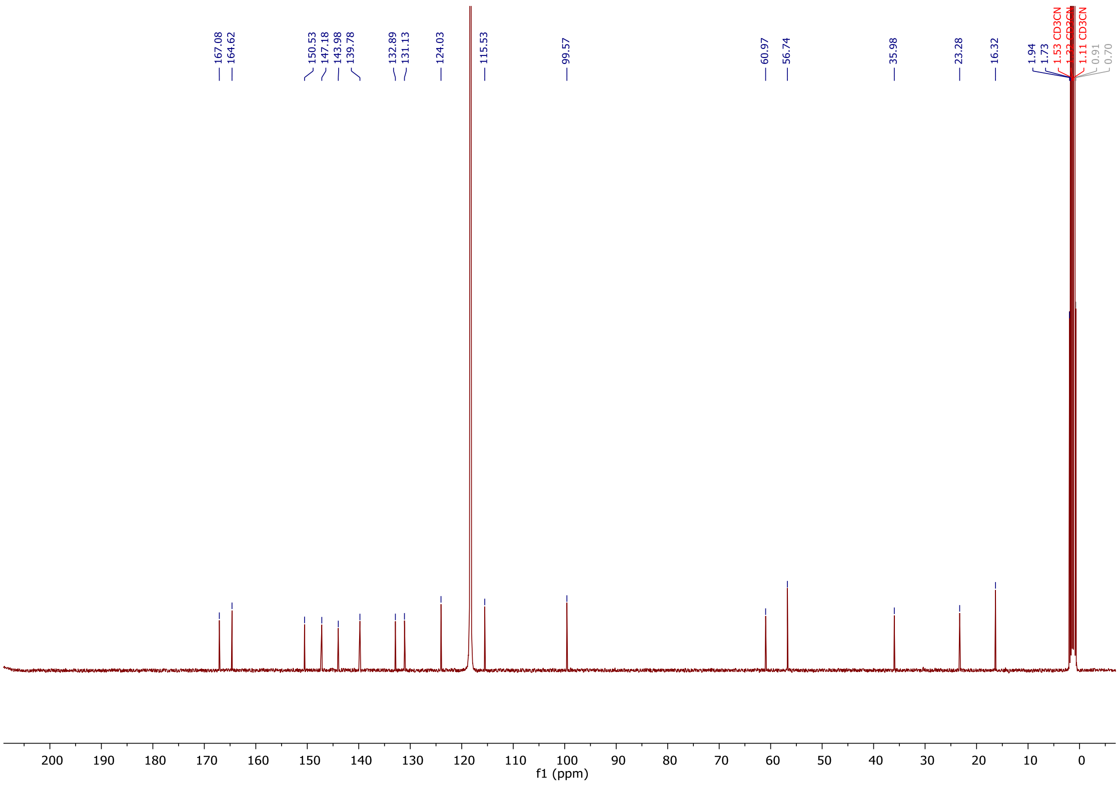

Romen
20220920_MS_22-0047 TSS 67 35 (1.497) Cm (33:36)	1: TOF MS ES+ 7.60e4

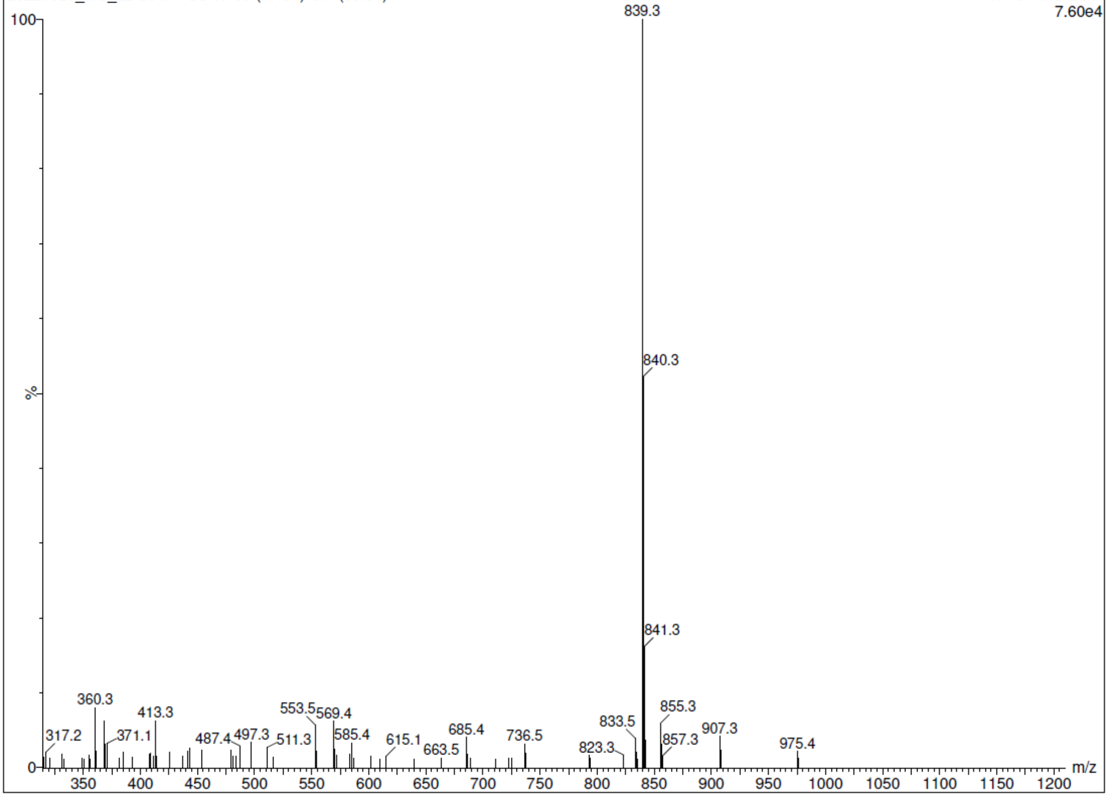

Elemental Composition Report

Page 1

Multiple Mass Analysis: 2 mass(es) processed

Tolerance = 5.0 PPM / DBE: min = -1.5, max = 100.0
Element prediction: Off
Number of isotope peaks used for i-FIT = 3

Monoisotopic Mass, Even Electron Ions
707 formula(e) evaluated with 4 results within limits (up to 50 best isotopic matches for each mass)
Elements Used:
C: 0-60 H: 0-80 O: 0-20 Na: 0-2

Romen
20220920_MS_22-0047 TSS 67 62 (2.682)	1: TOF MS ES+ 5.11e+003

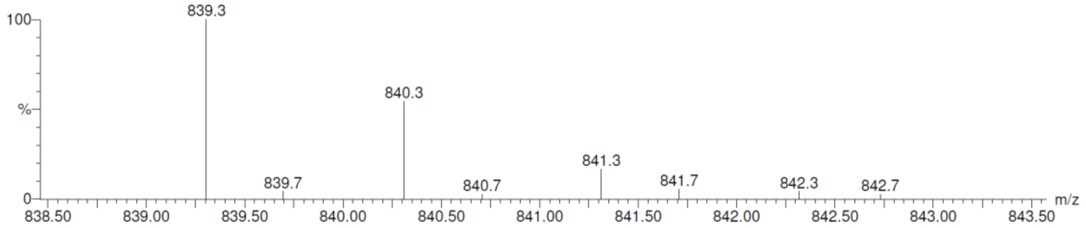

Minimum:	30.00				-1.5			
Maximum:	100.00		5.0	5.0	100.0			
Mass	RA	Calc. Mass	mDa	PPM	DBE	i-FIT	i-FIT (Norm)	Formula
839.3055	100.00	839.3043	1.2	1.4	24.5	39.6	0.2	C48 H48 O12 Na
		839.3068	-1.3	-1.5	27.5	41.7	2.3	C50 H47 O12
		839.3019	3.6	4.3	21.5	42.5	3.1	C46 H49 O12 Na2
		839.3078	-2.3	-2.7	12.5	46.9	7.5	C39 H53 O17 Na2
840.3088	54.35	---						

References

[1] Perretti, M. D.; Pérez-Márquez, L. A.; García-Rodríguez, R.; Carrillo, R. Building Covalent Molecular Capsules by Thiol-Michael Addition Click Reaction. *J. Org. Chem.* **2019**, *84*, 840–850.
